# Microbiota composition and intestinal integrity remain unaltered after the inclusion of hydrolysed *Nannochloropsis gaditana* in *Sparus aurata* diet

**DOI:** 10.1038/s41598-021-98087-5

**Published:** 2021-09-21

**Authors:** I. M. Cerezo-Ortega, D. E. Di Zeo-Sánchez, J. García-Márquez, I. Ruiz-Jarabo, M. I. Sáez-Casado, M. C. Balebona, M. A. Moriñigo, S. T. Tapia-Paniagua

**Affiliations:** 1 Department of Microbiology, Faculty of Sciences, CEI·MAR-International Campus of Excellence in Marine Science, University of Malaga, Málaga, Spain; 2 Department of Biology, Faculty of Marine and Environmental Sciences, CEI·MAR-International Campus of Excellence in Marine Science, University of Cádiz, Cádiz, Spain; 3 Department of Animal Physiology, Faculty of Biological Sciences, University Complutense, Madrid, Spain; 4 Department of Biology and Geology, CEI·MAR-International Campus of Excellence in Marine Science, University of Almería, Almería, Spain

**Keywords:** Applied microbiology, Microbial communities, Transcriptomics, Biotechnology, Microbiology, Molecular biology

## Abstract

The use of lysed microalgae in the diet of carnivorous fish can increase the bioavailability of proteins and bioactive compounds, such as unsaturated fatty acids or vitamins in the digestive tract. These are essential molecules for the proper physiological development of fish in aquaculture. However, some antinutritional components and other undesirable molecules can be released from an excess of microalgae supplied, compromising the integrity of the intestine. The inclusion of small amounts of hydrolized microalgae in the fish diet can be a good strategy to avoid negative effects, improving the availability of beneficial compounds. *Nannochloropsis gaditana* is an interesting microalgae as it contains nutraceuticals. Previous studies reported beneficial effects after its inclusion in the diet of *Sparus aurata*, a widely cultured species in Europe and in all Mediterranean countries. However, administration of raw microalgae can produce intestinal inflammation, increased intestinal permeability, bacterial translocation and disturbance of digestion and absorption processes. The aim of this study was to evaluate changes in the intestinal microbiota and barrier stability of *S. aurata* fed with low inclusion (5%) hydrolysed *N. gaditana*. Intestinal microbiota was analyzed using Illumina MiSeq technology and libraries were constructed using variable regions V3–V4 of 16S rDNA molecules. Analysis were based in the identification, quantification and comparison of sequences. The predictive intestinal microbial functionality was analyzed with PICRUSt software. The results determined that the intestinal microbiota bacterial composition and the predictive intestinal microbiota functionality did not change statistically after the inclusion of *N. gaditana* on the diet. The study of gene expression showed that genes involved in intestinal permeability and integrity were not altered in fish treated with the experimental diet. The potential functionality and bacterial taxonomic composition of the intestinal microbiota, and the expression of integrity and permeability genes in the intestine of the carnivorous fish *S. aurata* were not affected by the inclusion of hydrolysed 5% *N. gaditana* microalgae.

## Introduction

Research and improvement of commercial diets are crucial in aquaculture. The search for new sustainable products and the need to provide a better health status in aquaculture organisms are environmental priorities^1^. In order to ensure an optimal development or produce a beneficial effect on organisms, new functional diets are currently being developed in the aquaculture industry^1–4^.

Some strategies have been focused on the administration of probiotics, prebiotics, symbiotics, or its products, postbiotics^5^, and bioactive compounds^6–8^. The bioactive compounds are substances of natural origin with a high biological value, commonly found in terrestrial plants^9^ and algae^10,11^ because they are rich in proteins, essential amino acids, and polyunsaturated fatty acids^12^. Nutraceutical compounds usually exert immunomodulatory and growth-promoting effects^13–15^, reasons why their use has been proposed as a strong preventive strategy against opportunistic infections in the aquaculture industry and to improve cultured fish production^16^.

Various genera of microalgae are also important sources of bioactive compounds, such as sterols, proteins, enzymes, pigments, or vitamins^17–19^, One example is *Nannochloropsis,* which is a genus of robust, oleaginous microalgae that is considered a optimal candidate for commercial applications^20^. Specifically, *N. gaditana* (Lubian, 1982) is a promising algae for use in aquaculture. *N. gaditana* has a great photosynthetic efficiency and accumulates high quantities of carbon dioxide in the form of the lipids, such as omega-3^21^, eicosapentaenoic acid (EPA, C20:5n-3)^22^, and docosahexaenoic acid (DHA, C22:6n-3)^23^. Also, it is a great pigment source^24^, and contains aminoacidic and antioxidant compounds^25^. Its production is not expensive^22^, and it is used to feed fish, especially in their larval stages^26^.

However, the presence of a cell wall with cellulose can reduce in vivo bioavailability of nutrients and other intracellular compounds when the algae is incorporated to the feed^27,28^. This inaccessibility may also hide the expected positive results in the organisms. Four types of treatments to disrupt the algae cell wall to release their intracellular components have been described: enzymatic, chemical, physical and mechanical^29–31^. Each procedure provides different accessibility and modifications of the cellular components. Despite physical procedures are usually preferred as they provide greater digestibility while keeping the composition unaltered, enzymatic methods using enzymes with cellulase capacity have been successfully tested in culture fish and need to be considered^32^. In particular, enzymatic hydrolysis of microalgae cell wall may be a promising category since this process occurs under mild temperatures and reduce the possibilities of inhibiting by-products release^33^. However, the enzymatic hydrolysis process of the cell wall involves the risk of releasing intracellular substances that may cause undesirable physiological alterations to the fish^34^. The liberation of molecules from cellular lysis may affect the intestinal microbiota composition and functionality or increase inflammation, intestinal permeability, or bacterial translocation in the intestine^35^. For example, some microalgal species contain lectins, which are considered anti-nutritional elements^36,37^. However^38^, in gilthead seabream (*Sparus aurata*) juveniles fed with diets supplemented with *Arthrospira* enzymatic hydrolysates (2 and 4%), highlighted beneficial effects by describing increased activities of digestive enzymes such as trypsin, leucine aminopeptidase, or alkaline phosphatase, improving digestive processes and intestinal absorption capacity*,* and low inclusion of *N. gaditana* in *S. aurata* showed to be sufficient to obtain optimum growth without substantially increasing the cost of the feed^39^.

The mucus layer and the intracellular structures known as tight junctions play a major role in regulating the paracellular passage and permeabilization of luminal elements, such as nutrients, toxic molecules or infectious agents across the intestinal barrier. In fish, the permeability and integrity of this barrier is particularly affected by stress, changes in the microbiota, the presence of pathogens, changes in salinity, and diet^40^. However, the effects of algae hydrolysates on the taxonomic composition and functionality of intestinal microbiota and the stability of intestinal barrier have not yet been assayed. Those two aspects are implicated in digestion and absorption process, and closely related to the healthy status of fish. For these reasons, in this work, the analysis of the taxonomic composition and potential functionality of intestinal microbiota, and the measurement of the expression of genes related to the intestinal barrier stability in *S. aurata* fed with low inclusion of hydrolyzed *N. gaditana* have been carried out.

## Results

### Sequencing data analysis

Raw read sequences of the 16S rRNA gene from *S. aurata* gut microbiota in this study are publicly available in the NCBI SRA depository within BioProject PRJNA700500, with BioSample accession numbers SAMN17721432-SAMN17721459.

**Figure 1:**
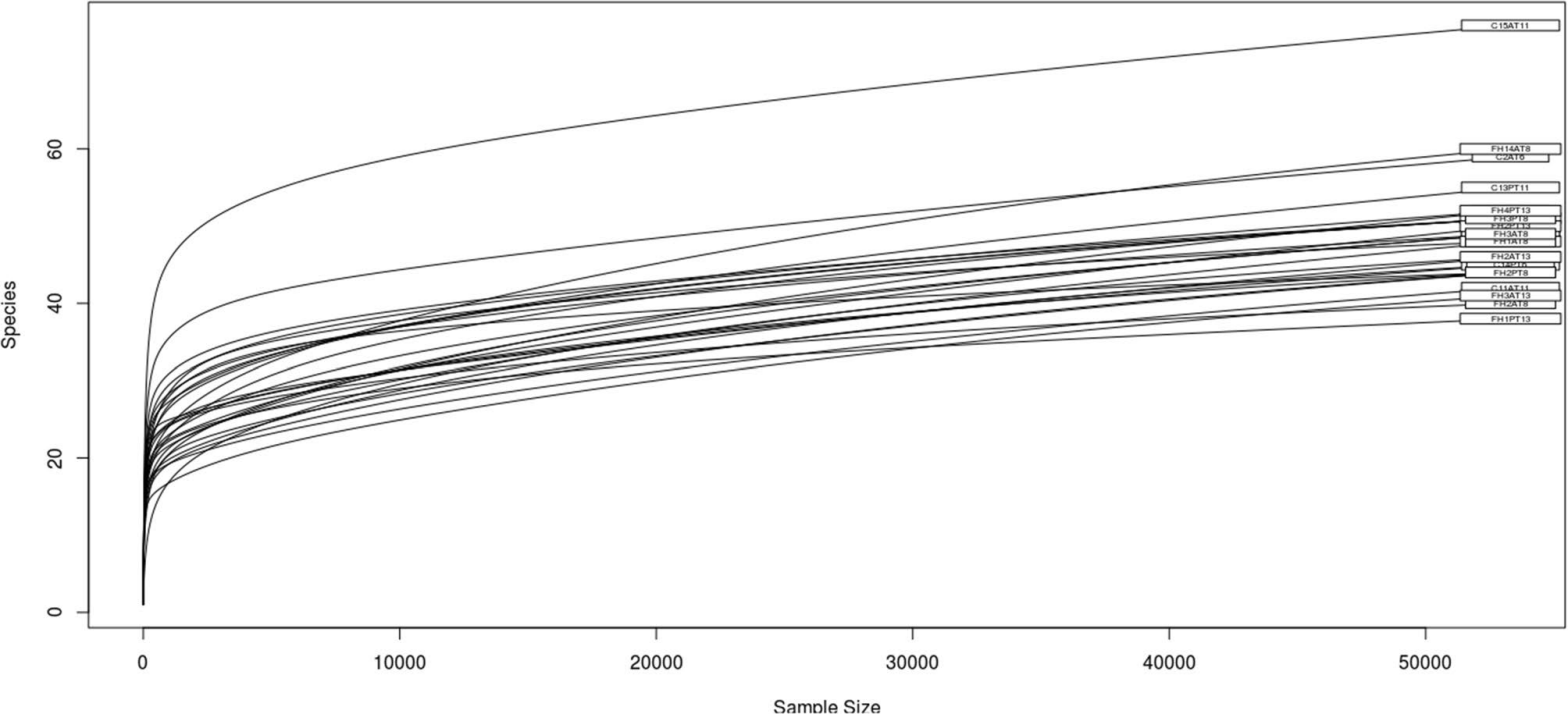
Rarefaction curves for each fish. Sequences were standardized to equal sample sizes for direct comparison.

Illumina Miseq sequencing yielded a total of 22,605,600 reads. After bioinformatic processing, a total of 74,653.78 ± 9905.93 readings per sample were obtained and classified into 261 OTUs. The data size was normalized to 53,370 reads, which corresponds to the minimum number of reads obtained in all the samples. The sequences were filtered through the rarefaction curves at 53,370 readings, thus leaving 245 OTUs. Sequence coverage was ≥ 99% in all cases (Fig. 1).

The medium number of OTUs, and Chao1, Shannon and Simpsom indexes were calculated for each sample of experimental groups (Table 1). The alfa diversity values in the two experimental groups and sections assayed did not show significant differences between the treatment Control-A and FH-A or Control-P and FH-P, where “A” means “anterior section ” and “P” “posterior section” of intestine.

**Table 1:** Ingredient composition of experimental diets used in the feeding trial.

Ingredients (g/kg DM)	Control diet	FH diet
Fishmeal LT^a^	200	200
*N. gaditana* meal	–	50
Squid meal^b^	20	20
Fish protein hydrolysate^c^	10	10
Krill meal^b^	20	20
Gluten meal^b^	150	150
Soybean protein concentrate^d^	366	335
Fish oil	114	105
Soybean lecithin	10	10
Maltodextrin	42	32
Lisin	12	12
Metionin	5	5
Choline chloride^e^	5	5
Vitamin and mineral premix	20	20
Guar gum^b^	10	10
Alginate^b^	10	10
Crude protein (%)	47.7 ± 1.5	46.6 ± 0.8
Crude lipid (%)	17.2 ± 0.5	16.5 ± 0.0
Ash (%)	5.3 ± 0.3	5.4 ± 0.1

FH diet contains 5% hydrolysed raw algal biomass.

^a^Norsildemel (Bergen, Norway).

^b^Lifebioencapsulation (Almería, Spain).

^c^(81% crude protein, 8.8% crude lipid) Sopropeche (France).

^d^(65% crude protein, 8% crude lipid) DSM (France).

^e^Sigma-Aldrich (Madrid, Spain).

### Taxonomic composition of the intestinal microbiota

**Figure 2:**
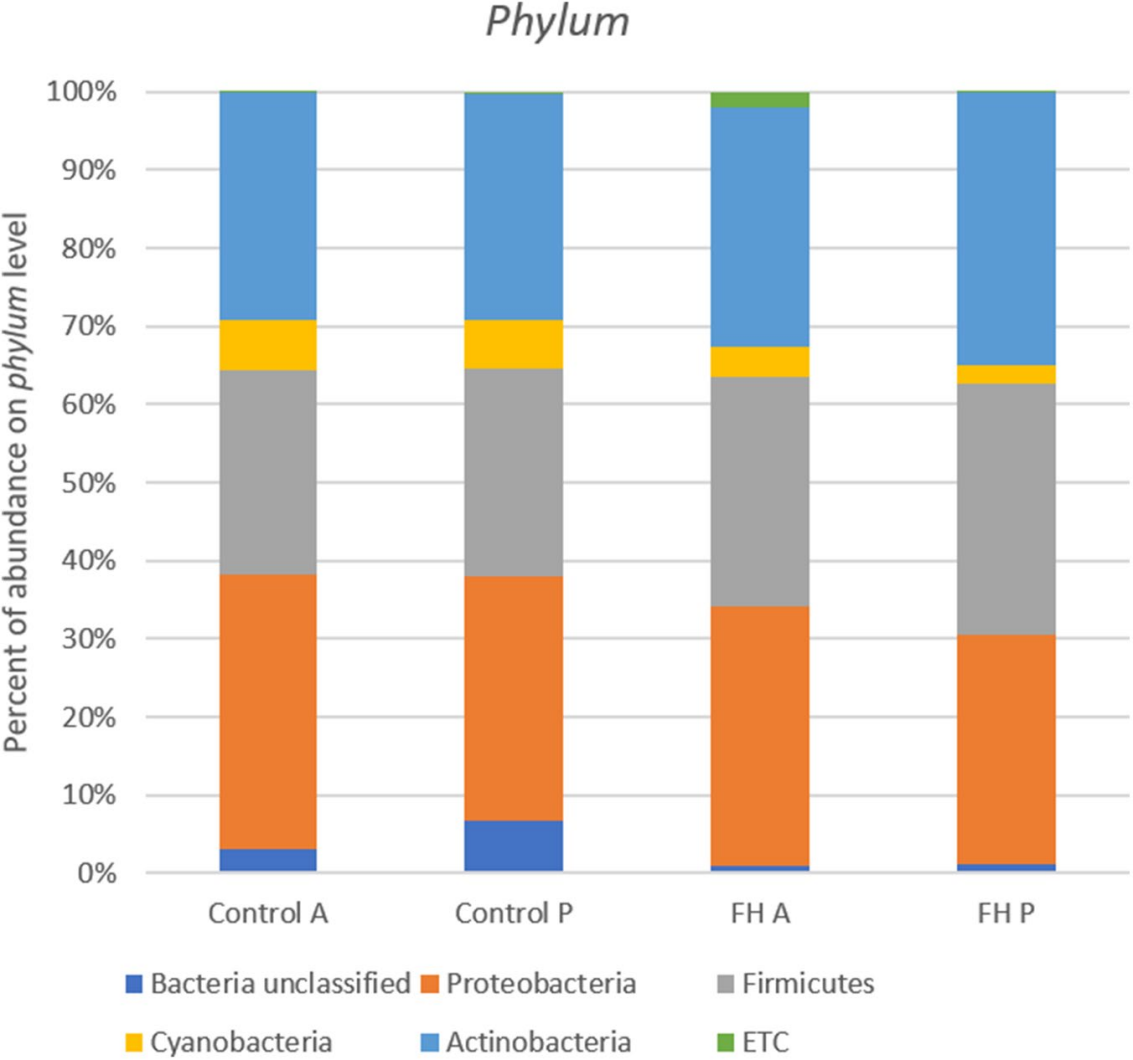
Comparison of microbial communities at *phylum* level obtained for each diet, Averages were calculated for intragroup. Control A and Control P are referred to the Control diet in the anterior (A) and posterior (P) sections of the intestine. FH (number)-A and FH(number)-P are referred to the supplemented diet with *N. gaditana* in the anterior and posterior sections of the intestine. ETC: *phylum* represented < 1%.

The predominant *Phylum* detected in all fish fed with two diets were Proteobacteria, followed by Firmicutes, Cyanobacteria and Actinobacteria, and although the quantity of them were slightly different in function of the diet administrated, no significant differences were found among diets or intestinal sections (Fig. 2).

**Figure 3:**
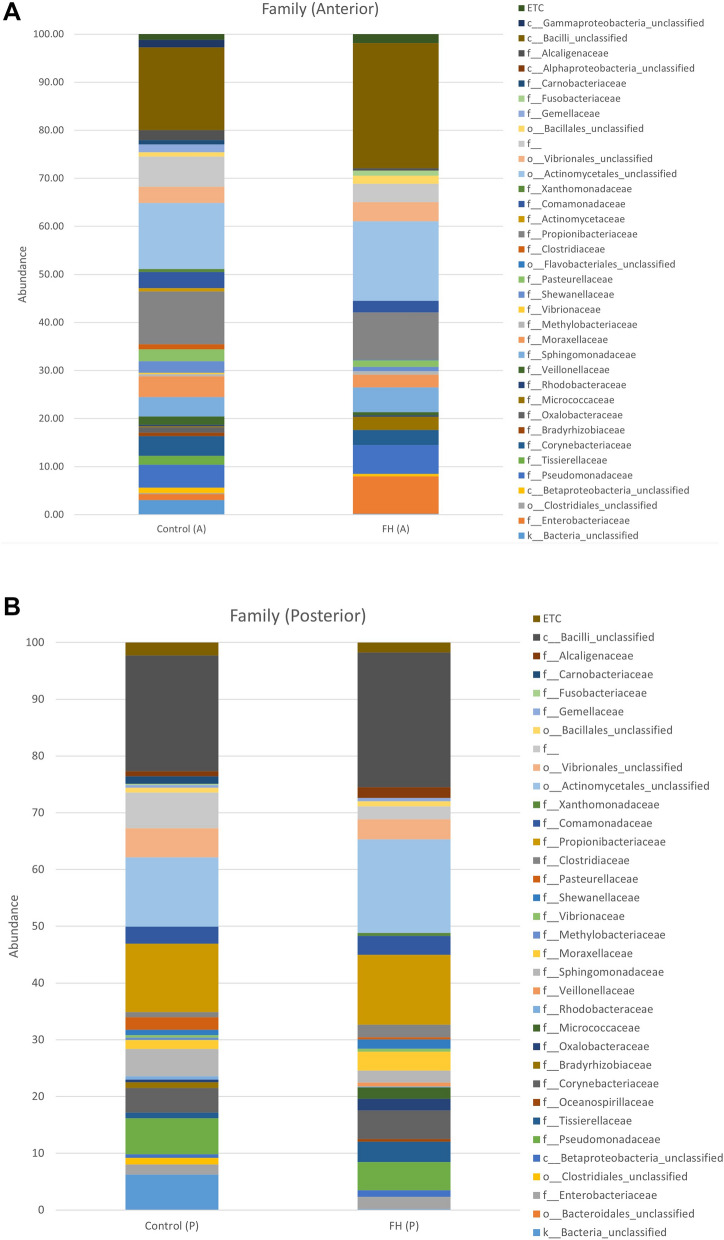
Average values of bacterial family groups obtained in the intestinal microbiota samples in anterior (A) and posterior (P) sections (**A**,**B**, respectively) of *S. aurata*. Control (C-number) and *N. gaditana* hydrolyzed (FH-number) diets. ETC: families represented < 1%.

The bacterial families and group found in the anterior section of specimens were heterogeneous in all the groups. In this section of the intestine, *Enterobacteriaceae*, *Pseudomonadaceae*, *Sphingomonadaceae*, *Propionibacteriaceae*, *Fusobacteriaceae* and *Actinomycetales*, *Vibrionales,* and *Bacilli,* which were not identify to family level, its average values increase in FH-A compared to Control-A, but not significantly. *Clostridiaceae, Gemellaceae, Carnobacteriaceae* and *Tissierellaceae* were not present in the treatment FH-A, but they are present in Control A. *Fusobacteriaceae* was no present in Control-A but it appears in FH-A (Fig. 3A). In the posterior section, *Moraxellaceae*, *Vibrionaceae*, *Actinomycetales*, and *Bacilli_unclassified* increased, but *Enterobacteriaceae and Pseudomonadaceae* decreased in FH-P. *Carnobacteriaceae* family and *Clostridiales* order disappeared in FH-P treatment. Statistically significant differences were not detected in any case and data have been represented to show the variation between treatments (Fig. 3B).

**Figure 4:**
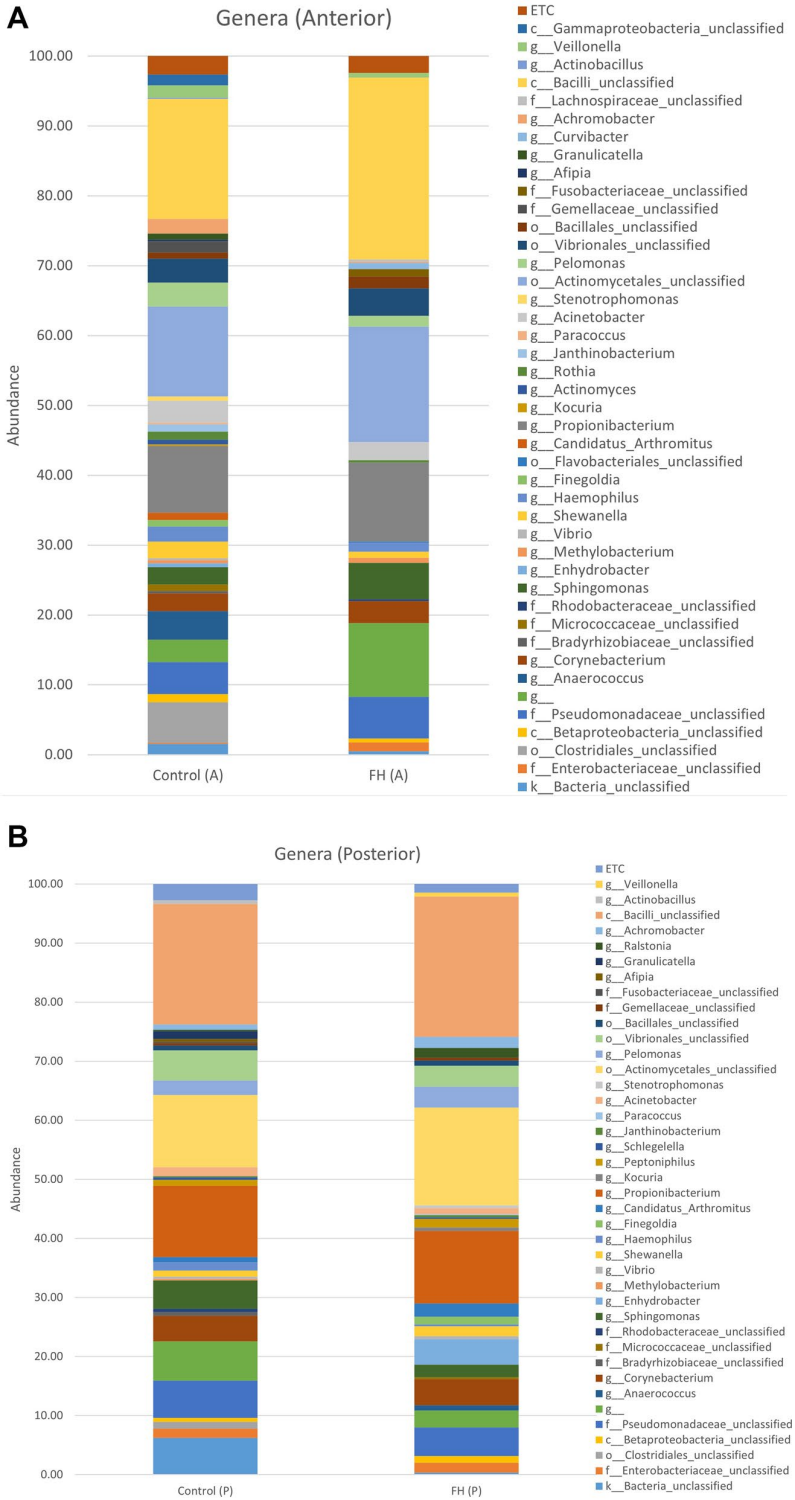
Average values of bacterial genera groups obtained in the intestinal microbiota samples in posterior (P) and anterior (A) sections (**A**,**B**, respectively). Control (C-number) and *N. gaditana* hydrolyzed (FH-number) diets. ETC: genera represented < 1%.

Some genera were detected between diets As an example, in the anterior section some genera like *Clostridiales* or *Anaerococcus* are observable in Control-A but not in FH-A (Fig. 4A). In the posterior section, *Enhydrobacter*, *Actinomycetales* or *Bacilli*_unclassifiedincreased their average values in the FH-P treatment, but *Vibrio* and *Sphingomonas* decreased in this group.(Fig. 4B).

**Figure 5:**
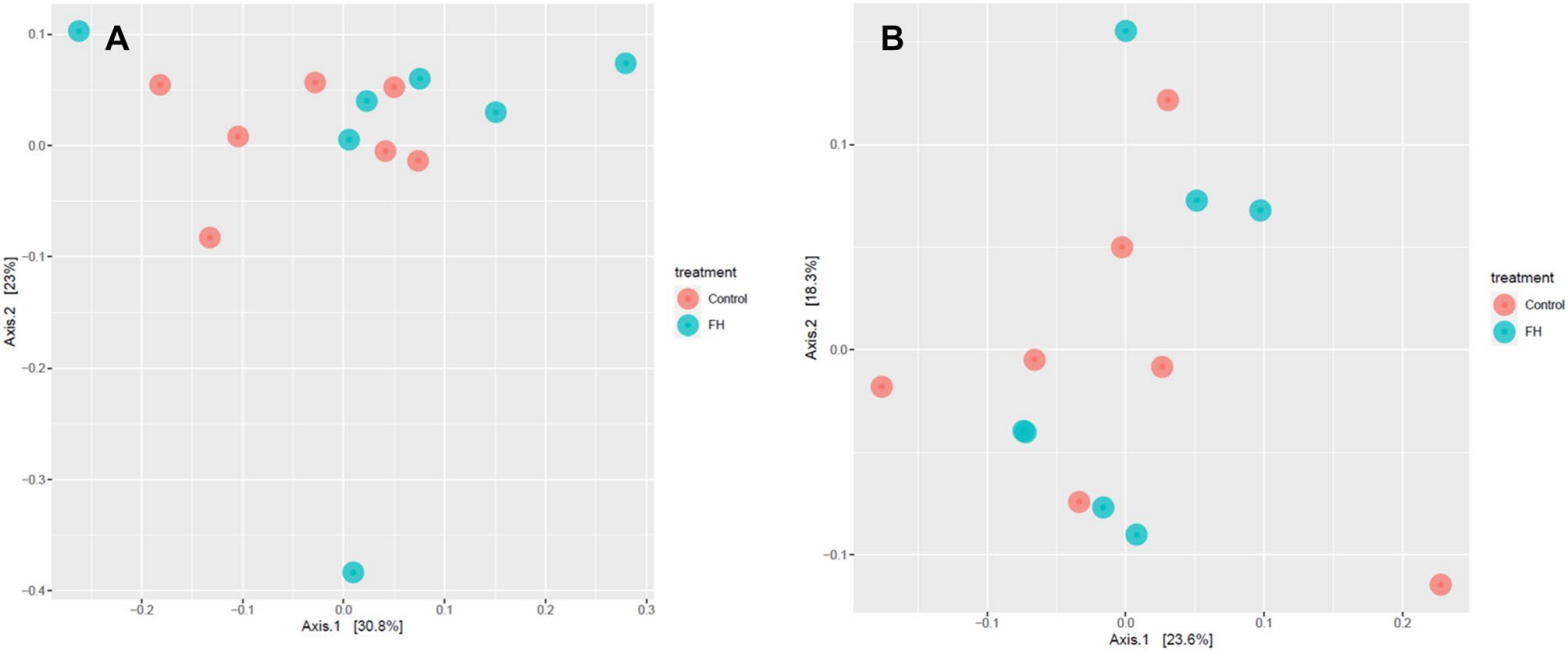
Principal principal coordinate analysis (PCoA) of the similarity of microbiota (OTUs) in anterior and posterior sections (**A**,**B**, respectively) of each individual of *S. aurata* fed with a Control or *N. gaditana* hydrolyzed (FH) diets.

Principal coordinates analysis (PCoA) scores are plotted based on the relative abundance of OTUs of intestinal microbiota from analyzed specimens. Each point represents a single sample, and the distance between points represents how compositionally different the samples are from one another. The points did not show a clear differences in the microbial community composition between control and FH diet (Fig. 5).

### Predictive study about the intestinal microbiota functionality

The functional study executed by PICRUSt software presented seven principal levels (metabolism, genetic information processing, environmental information processing, cellular processes, organismal systems, human diseases and drug development), but only three of them related to intestinal functions have been independently studied (metabolism, cellular processes and environmental information processing). Results obtained show that these functions were not affected by FH diet.

**Figure 6:**
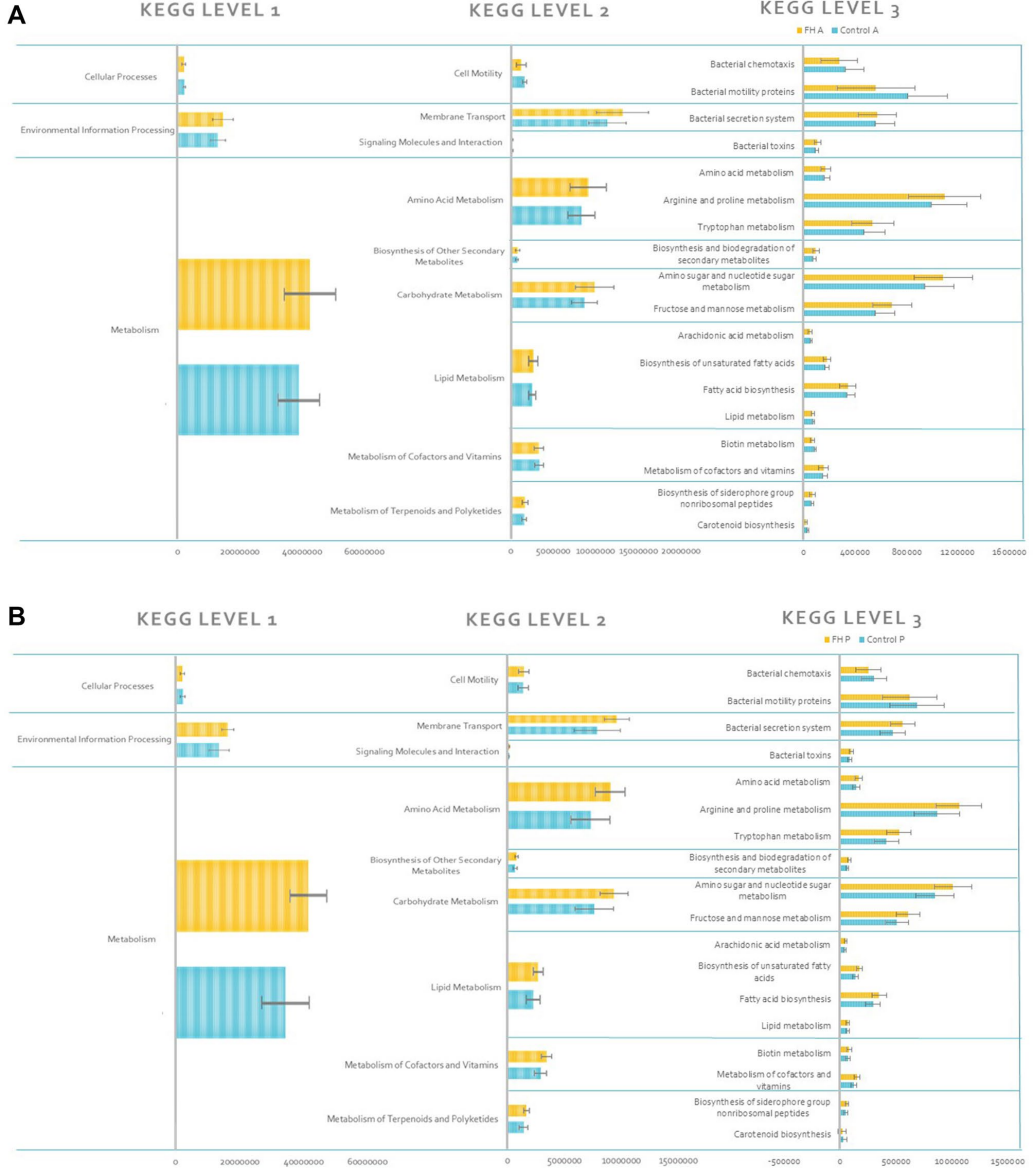
PICRUSt predictions of the functional composition of *S. aurata* gut microbiota. (**A**) Represents KEGG pathway at level 1,2 and 3, in anterior section and (**B**) in posterior section. Blue and yellow bars make reference to Control and FH diets, respectively.

The three functions analyzed (metabolism, cellular processes and environmental information processing) did not change between treatments in the KEGG 1 category, although the processes related to environmental information processing and cellular processes were increased lightly in FH-A (Fig. 6A) and FH-P (Fig. 6B) treatments, but without statistical differences.

In KEGG 2 category, functions had similar values between treatments, except in the carbohydrates and amino acid metabolism that increased in FH-A (Fig. 6A) and FH-P (Fig. 6B), but statistical differences were not found. Statistical differences were not found between intestinal sections in any studied KEGG 3 category.

### Gene expression

**Figure 7:**
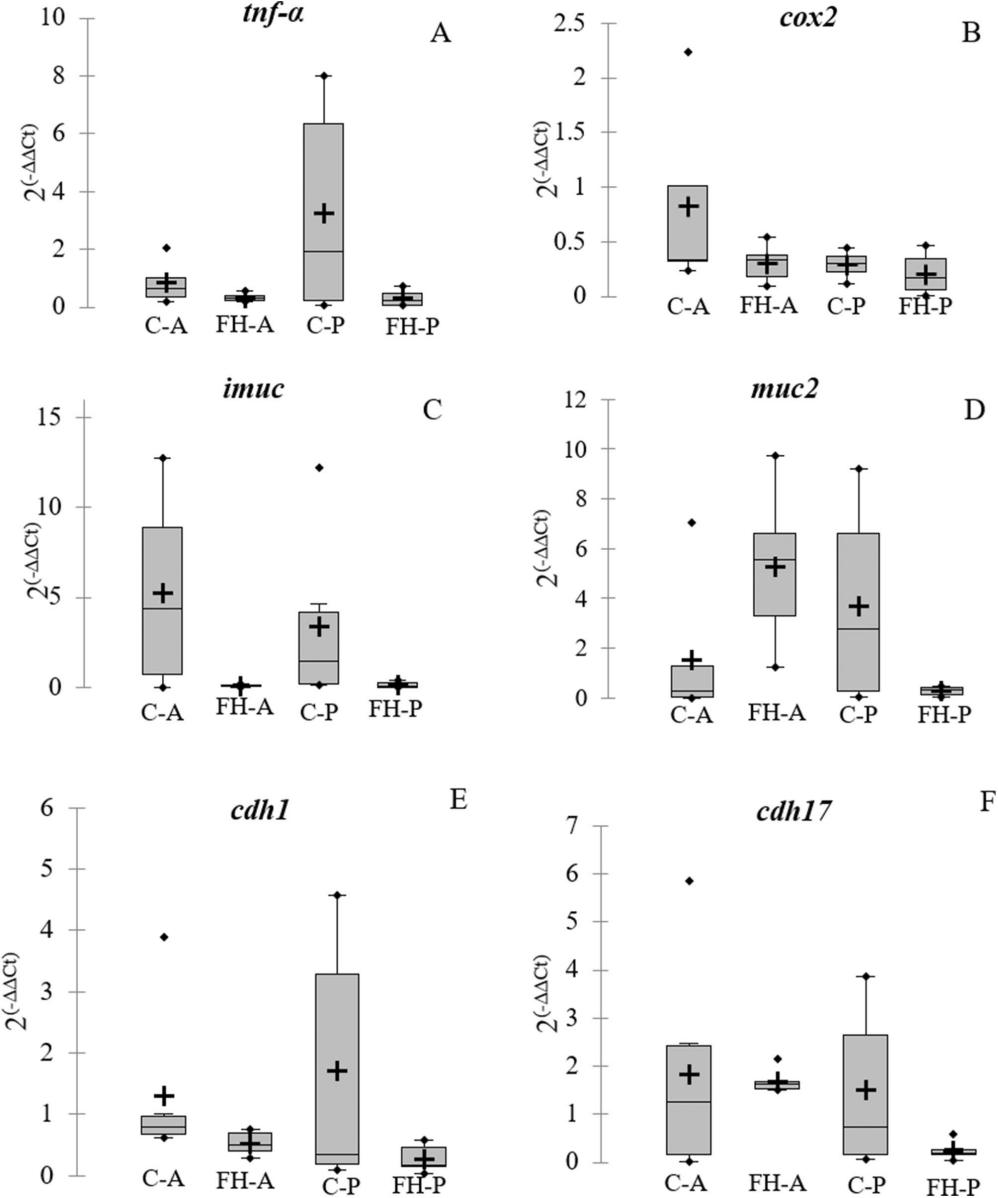

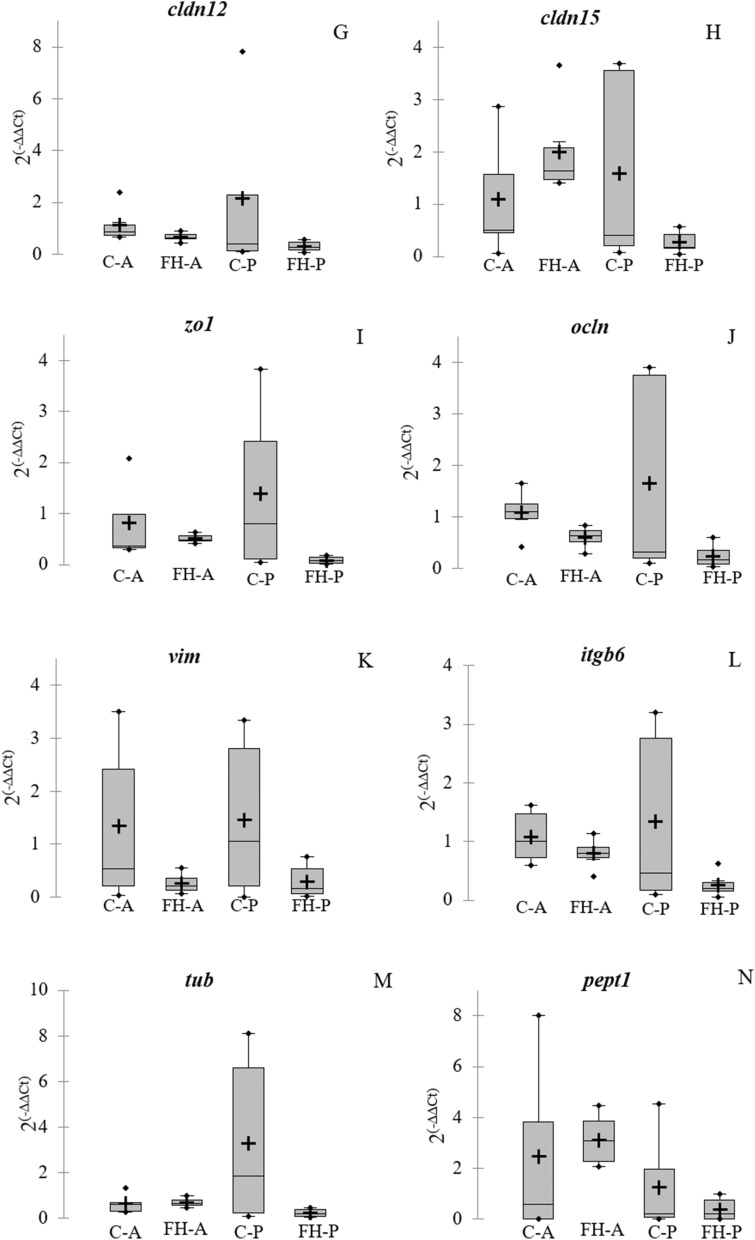
Boxplots for relative gene expression in the intestine of gilthead seabream fed with the experimental diet (FH) and the control group (C). (**A**) necrosis tumoral factor α (*tnf-α*); (**B**) cycloogygenase 2 (*cox2*); (**C**) intestine mucin (*imuc*); (**D**) mucin 2 (*muc2*); (**E**) cadherin 1 (*cdh1*); (**F**) cadherin 17 (*cdh17*); (**G**) claudin 12 (*cldn12*); (**H**) claudin 15 (*cldn15*); (**I**) zona-occludens 1 (*zo1*); (**J**) ocludin (*ocln*); (**K**) vimentin (*vim*); (**L**) integrin 6-β (*itgb6*); (**M**) tubulin (*tub*) and **N**, peptide transporter 1 (*pept1*). Bars represent relative gene expression (mean + standard error) for each group, in the intestinal anterior section (A) and the posterior section (P). The crosses correspond to the means and the horizontal bars to the median. Points above or below the upper and lower limits are considered outliers. Differences between experimental groups in each section were considered at *p* < 0.05.

In general, the FH group data showed less dispersion in the gene expression data than the control individuals. In addition, a trend was observed on down-regulation of the genes involved in the intestinal permeability, especially in the posterior section, and in the pro-inflammatory response in both intestine sections (*p* > 0.05) (Fig. 7A–M). Non-significant slightly up-regulation was also observed in the genes *pept1* and *muc2* related to nutrition and intestine protection, respectively, in the anterior section in fish receiving the FH diet (*p* > 0.05) (Fig. 7D,N). However, no significant differences were observed in the relative expression of the genes analyzed in the intestinal samples of fish fed with control or FH diets (Fig. 7).

## Discussion

The present study highlights that the carnivorous fish *S. aurata* fed with 5% of hydrolyzed microalga *N. gaditana* did not modify its intestinal microbiota after 12 weeks. This could result in an advance in the formulation of fish diets as microalgae are important sources of essential compounds and beneficial nutriceuticals.

In the present study, the tendency of more homogeneous data (less values of variance) and medium values of diversity index between the anterior and posterior sections have been observed when the fish are fed with the FH diet. Reference^41^ concluded that changes in diversity are due to an adaptation of the microbiota to digest and assimilate the ingredients added, such as cell walls, polysaccharides and lipids, which affect or modify colonization by minority groups. Avoiding loss of diversity is considered as a positive aspect that protects intestinal mucose and gives protection against diseases^42^. Likewise, authors have established that microbial diversity is considered a biomarker for fish health and an image of a good metabolic capacity^43^ and recently^44^, affirm that *S. aurata* have a plastic microbiota which effectively adapts to the metabolic challenges induced by dietary changes. The same authors have proposed studies focused on how these microbial changes correlate with health, growth, and disease resilience.

Previous studies carried out by Ref.^26^ about the effect of *N. gaditana* on seabream’s intestinal microbiota have not shown alterations in intestinal morphology and function with the same percentage of inclusion as in the present study, but the duration of the trial was limited to 1 month and *N. gaditana*’s entire cells were used. The *phylum* detected in this work, Actinobacteria, Firmicutes and Proteobacteria are part of the normal microbiota in *S. aurata*^45–47^. Some groups such as *Micrococcaceae* and *Ralstonia* present antimicrobial activity, biosynthesis of bioactive compounds and can produce beneficial secondary metabolites for the host^48^, and they have been detected in the intestinal microbiota of the FH group. *Micrococcaceae* family contain enzyme producing bacteria capable of producing amylases, cellulases, proteases, lipases, phytases, tannases, xylanase and chitinase^49^. These enzymes can contribute to the digestion and assimilation of algal products. According to Ref.^50^ members of Actinobacteria group are butyrate-producing bacteria. Butyrate is a short-chain fatty acid (SCFA) with important and demonstrated beneficial effects, also in fish such as *S. aurata*^51^. In this study Actinobacteria have been detected but the fine representation of all the members of this group may be minor to 1% and they are incluyed in ETC group (0.1% *Streptococcu*s, or 0.6% *Actinomyces*, among others).

In the posterior section, FH group showed a low increase in the presence of Actinobacteria and a decrease in Cyanobacteria, but not statistically significant. Cyanobacteria in general produces lipopolysaccharides that are inflammatory agents and gastrointestinal irritants^52^ and causes damage in the host by facilitating the colonization of other dangerous bacteria^53,54^.

*Clostridiaceae* is a family that appeared in FH-P group. Those group is related to dysbiosis in human^55^ but in other animals such as pigs, *Clostridiaceae* has been related to improvements in feed efficiency and growth performance, being alsoconsidered beneficial to fish^44^. FH-A group showed *Bacilli* and *Actinomycetales*. Those groups are present when the diet is enriched in vegetals^56^.

Some genera weredetected in the present study were significantly different between samples. In the anterior and posterior sections it was observable genera like *Clostridiales* in Control-A but was not present in FH-A. In the posterior section, *Enhydrobacter* appearedin the treatment FH-P. *Enhydrobacter* is present in the microbiota of fish such as *Dicentrarchus labrax* and *Salmo trutta*^57^*.* It is known that this genus utilizes certain amino acids such as l-arginine, l-serine and l-alanine^58^, while some species present cellulase activity^57,59^. This fact may be of interest as cellulases hydrolyze the cellular wall of *N. gaditana*, allowing the fish to absorb the intracellular compounds of this algae. Thus, the intestinal microbiota of *S. aurata* is able to adapt to dietary changes, as proposed before^44^.

By a taxonomical description of the bacterial groups present in the intestinal microbiota the information about their function at the gut is unknown^60^. The disponibility of a predictive software allows us to relate the sequences obtained with the OTUs and the predominant functions going on at the intestine. In this study, no differences were found in any of the seven principal functional pathways. The three functional pathways analyzed, metabolism, cellular processes and environmental information processing represented 63.12 ± 0.83% of total functional enrichment per sample. Additionally, KEGG 2 and 3 levels were analyzed but none of them were statistically different between treatments with the exception of a slight increasing trend detected in the FH treatment (but not statistically significative). It is important to point out that the inclusion of hydrolyzed *N. gaditana* does not increase the presence of functions related to bacterial toxin synthesis. The rest of the functions were represented in the same quantity. The hydrolyzed cells do not affect negatively the bacterial functions, studied by predictive form.

In other studies with *S. aurata* juveniles, it was observed that low dietary supplementation with *Arthrospira* hydrolysates (2 and 4%) had no effects on the intestinal mucosa^38^. Similar results were found by Saenz et al. (personal communication/unpublished results) in *S. aurata* within the present study. In this sense, the results reported by a visual analysis of images obtained by transmission electron microscopy (TEM) and scanning electron microscopy (SEM) have not revealed signs of damage or irritation in the intestinal microvilli. In this regard, *N. gaditana* in the diet of *S. aurata* does not alter neither the intestinal microbiota composition nor its functions, significantly. Our in vivo results support previous information described in vitro by Ref.^32^, where *N. gaditana* added to *S. aurata* intestinal extract lead to an improvement in the digestion and assimilation of nutrients^61^.

The inclusion of 5% *N. gaditana* in the diet has not produced any significant alteration in the expression of genes involved in the enterocytes cell-to-cell adhesion, the cytoskeleton and the epidermal cell-basement membrane adhesion (*cdh1*, *cdh17*, *cldn12*, *cldn15*, *vim*, *itgb6*, *ocln*, *tub,* and *zo1*) and, therefore, it is very likely that the integrity and permeability of the analyzed fish remain unaltered. This information is further supported by the absence of changes in the expression of markers commonly used to measure pro-inflammatory processes (*tnf-α* and *cox2*), which have been associated with increased permeability and intestinal disruption in several studies^35,45,62,63^. The absence of statistically significant differences in our study could also be due to a high dispersion between individuals. However, a tendency on a down-regulation of the tight-junction genes analysed was observed in the FH group. Experimental diets that have shown to impair fish growth have also been related to a reduction in the expression of genes involved in maintaining tight junctions^45^ whereas their upregulation has been associated with a healthier intestinal barrier^7^. Contradictorily, a gene up-regulation of tight junctions in response to various experimental diets has also been related to nutritional, osmoregulatory and physiological alterations, thus indicating an undesirable state in gilthead seabream^51,64^. Therefore, further analysis of immune system-related genes, as well as histological studies are necessary to confirm the actual status of the gut integrity and permeability.

The peptide transporter 1 (PepT1) is a low-affinity/high-capacity nutrient transporter that mediates the uptake of dipeptides and tripeptides from diet, which are essential for fish to sustain development, growth and metabolism^65^. In our study, no statistically significant difference was observed in *pept1* gene expression between the groups analyzed. However, both Control and FH groups showed higher expression levels of *pept1* in the anterior intestine section compared to the posterior section. Additionally, a slight up-regulation of *pept1* was observed in the FH group compared to the control group in the anterior intestine section. Higher *pept1* expression in the anterior section is consistent with studies indicating that the proximal intestine is the area of highest expression of this protein in fish, specifically at the absorptive epithelial cells along the mucosal folds^66,67^. The trend indicating a slight up-regulation of *pept1* in the FH group may suggest improved nutrient absorption. In higher vertebrates, more weight gain and increased expression of *pept1* in the intestine were found in response to a high-quality soybean protein diet^68^. Reference^62^ found that a diet containing 15% of vegetable proteins (green pea protein concentrate) induced significantly lower levels of *pept1* transcripts and were associated with lower growth in gilthead seabream^67^. Gilthead seabream fed on a strict vegetable protein diet also showed lower expression levels of *pept1*, and has been related to a lower digestibility and small peptide transport^69^. Taking *pept1* gene expression as a marker of the dietary protein quality and absorption efficiency, our results suggest that the substitution of 5% of *N. gaditana* in the diet is probably not affecting the nutrient intake in gilthead seabream, or that the high dispersion between individuals may be affecting the significance of the results and, therefore, hiding possible beneficial effects.

Mucins contribute to protect the intestine epithelium against a broad spectrum of damages^70^. Enhanced gut mucin production has been related to reduced bacterial translocation in fish fed with a diet containing yeast-derived oligosaccharides^63^. In this study, the inclusion of *N. gaditana* did not affect the mucins related genes (*muc2* and *imuc*) between the Control and FH groups.

In summary, the inclusion of 5% *N. gaditana* microalgae subjected to enzymatic hydrolysis in the commercial diet does not alter the composition and functionality of the intestinal microbiota, nor the expression of integrity and permeability genes in the intestine of the carnivorous fish *S. aurata.*

## Materials and methods

### Feed preparation

The *N. gaditana* biomass was produced in closed tubular photobioreactors in *La Estación Experimental de las Palmerillas* (Fundación Cajamar, Almería, Spain) as reported by Ref.^71^. Raw microalgae paste at provided were collected and immediately used for enzymatic hydrolysis according to a previous described protocol^38^. Briefly, *N. gaditana* sludge containing up to 150 g L^−1^ of raw microalgae biomass was hydrolyzed using commercial enzymes with cellulase activity (Viscozyme^®^) under controlled conditions (pH 5.0 and 50 °C under continuous stirring) for 4 h, providing 2% (w/w) enzyme. The experimental aquafeed was elaborated at the CEIA3-Universidad de Almería facilities (Servicio de Piensos Experimentales, http://www.ual.es/stecnicos_spe) including 5% of the hydrolyzed algae (FH diet). The microalgae concentrations employed were based on previous studies^39^. The solids were mixed in a kneader and, subsequently, the resulting mixture was extruded to obtain granules with the desired diameter (2–3 mm). Feed was stored at −20 °C until use. Control feed composition is showed in Table 1.

### Experiment design and sampling

The feeding assay was carried out at *Servicios Centrales de Investigación en Cultivos Marinos* (SCI-CM, CASEM, University of Cadiz, Puerto Real, Cádiz, Spain; Facilities for Breeding, Supplying and Users of Experimental Animals; Spanish Operational Code REGA ES11028000312). In the trial, juveniles of gilthead seabream of approximately 17.08 ± 0.38 g and 10.2 ± 0.1 cm fork length (mean ± SEM) were randomly distributed in 6 tanks of 80 L capacity (N = 15 fish per tank). The experiment was divided into two different fed conditions, one receiving a commercial diet (control group) and the other receiving a diet supplemented with 5% of fresh hydrolyzed *N. gaditana* paste (FH group) for 86 days. At the end of the growth assay, the animals (fasted for 12 h) were netted and deeply anesthetized with 2-phenoxiethanol (1 mL/L, Sigma-Aldrich 77699), and intestinal samples from seven fish per treatment (three fish from one tank, and two fish from the other two tanks) were extracted. Then, each tract was divided into two major sections, anterior and posterior sections, and kept at −80 °C until use. Remaining fish from each tank were employed in a parallel study (unpublished results).

### DNA extraction and sequencing by Illumina Miseq technology

DNA of both intestinal sections were extracted according to the protocol described by Ref.^72^ based on saline precipitation, with modifications^73^. Briefly, the samples were mixed with 300 µL of resuspension buffer (0.1 M Tris–HCl, 0.01 M NaCl, 0.1 M EDTA, pH 8) and 300 µL of lysis buffer (0.1 M Tris–HCl, 0.1 M EDTA, 0.01 M NaCl, 1% SDS, pH 8.0), gently inverting the tube to mix thoroughly. The samples were treated with 32 µL NaCl 6 M and proteinase K (150 μg/mL) at 55 °C for 2 h, and lysozyme (10 mg/mL) at room temperature. Next, 6 M NaCl was added to reach a final concentration of 1.5 M. The solution was chilled on ice for 10 min followed by centrifugation at 13,000 rpm for 3 min. The clear supernatant containing genomic DNA was transferred to another tube containing an equal volume of isopropanol. The tubes were inverted gently several times. The DNA was pelleted by centrifugation at 13,000 rpm for 3 min. The DNA pellet was then washed in 70% ethanol. The dried DNA pellet was resuspended in 100 μL of TE buffer (10 mM Tris–HCl, 1 mM EDTA, pH 8.0) and stored at 4 °C. DNA quality and integrity were visualized by electrophoresis in 1% agarose gels, stained with GelRed Nucleic Acid Stain 20000x (InTRON Biotechnology, Seoul, Korea). Concentration was determined by using Qubit 2.0 fluorometer (Thermo Fisher Scientific, Germany). DNA was stored at −20 °C for further processing and 30 ng were used for subsequent analyses.

Libraries were constructed by the Ultrasequencing Service of the Bioinnovation Center (University of Malaga, Spain) using the Illumina MiSeq Platform. Sequencing was carried out using forward (5′ TCGTCGGCAGCGTCAGATGTGTATAAGAGACAGCCTACGGGNGGCWGCAG 3′), and reverse (5′-GTCTCGTGGGCTC GGAGATGTGTATAAGAGACAGGACTACHVGGGTATCTAATCC-3′) primers targeting the V3–V4 variable regions of the 16S rRNA gene.

A workflow based on the MOTHUR program (version 1.39.5) was used to remove Illumina adapter sequences and demultiplexing. The reads were filtered excluding reads < 80 bp or > 2000 bp long. Besides, the singleton sequences and the chimeras were discarded by UCHIME version 4.2 (https://drive5.com/uchime). Non-specific PCR amplicons were eliminated. The remaining representative, non-chimeric sequences were then subjected to taxonomic assignment against the Greengenes 16S database (May 2013), with 97% 16S similarity as the cutoff and clustered into Operational Taxonomic Units (OTUs).

After generating the taxonomic profile of microbiome samples, a comparison of taxa present in the samples was carried out. The data size was normalized to the minimum number of reads obtained in all the samples. All statistical analyses were performed using statistical software R and a web tool MicrobiomeAnalyst^74^. To determine the level of sequencing depth, rarefaction curves were obtained by plotting the number of observed OTUs against the number of sequences and Good’s coverage coefficient calculated. Alpha diversity was estimated based on Shannon, Chao1, and Simpson indexes to assay taxonomic and phylogenetic structure diversity, respectively. The results are presented at phylum, family and genus taxonomic levels. The group with relative abundance less than 1% have been considered “ETC” according to Ref.^75^ where'as taxa constituting ≥ 1% of the total number of cells' and 'rare phylotypes': as taxa constituting ≤ 0.1% of the total number of cells'. This limit is defined based on the levels that can be detected with PCR-dependent techniques^76^. We have used 1% because this is the limit of detection in molecular techniques such as DGGE, which we have used in the past to study intestinal microbiota^73,77–79^. Non-parametric Kruskal–Wallis statistical test was performed. The differences were considered statistically significant assuming *p* < 0.05. Finally, a multivariate analysis of OTU data was performed via Principal Coordinate Analysis (PCoA) of OTU profiles using Bray Curtis metric to represent differences between microbiota of each group. PCoA are plotted against each other to summarize the microbial community compositional differences between samples.

### Functional profiling of microbial communities

Predictive microbiota functions were made using PICRUSt (version 1.1.3), a tool designed to infer metagenomics information from 16S rRNA amplicon sequencing data^80^. The resulting metagenomics data were entered into the Greengenes database (version 13.5), and the metagenome prediction of bacterial communities was conducted using the calculated dataset after normalizing for DNAr16S copy number. Nearest Sequenced Taxon Index (NSTI) scores for evaluating the accuracy of predicted metagenomes were categorized with the Kyoto encyclopedia of genes and genomes (KEGG) pathways database^81^. Bacterial functional profiles until KEGG modules level 3 were compared, and the STAMP (Statistical Analysis of Metagenomics Profiles) was used to analyze the differential abundance of modules by intestinal sections and diet using ANOVA multiple-comparison test with post hoc Tukey- Kramer (*p* < 0.05).

### RNA isolation and quantitative PCR (qPCR)

In order to assess the gene expression throughout the intestinal tract, RNA was isolated from each intestine section, following TRIsure™ (Bioline, England) manufacturer instructions. RNA quantity was determined in Qubit (Thermo Scientific) and reverse transcription was performed using First Strand cDNA Synthesis Kit (Thermo Scientific) with 500 ng of total RNA. One microliter of each cDNA was employed as the template in the qPCRs to analyze each gene transcription. Specific primers were used for the quantification of the relative gene expression genes involved in intestinal permeability and integrity, such as cadherin 1 (*cdh1*), cadherin 17 (*cdh17*), claudin 12 (*cldn12*), claudin 15 (*cldn15*), vimentin (*vim*), integrin 6-β (*itgb6*), ocludin (*ocln*), tubulin (*tub*) and zona-occludens 1 (*zo1*); pro-inflamamtory reactions and mucins production, such as tumoral necrosis factor α (*tnf-α*), cycloogygenase 2 (*cox2*), intestine mucin (*imuc*) and mucin 2 (*muc2*); and nutrient absorption, such as the peptide transporter 1 (*pept1*) (Table 2, Supplementary Information). For normalization, the samples were analyzed in parallel with two reference genes, elongation factor 1α (*ef1α*) and ribosomal glyceraldehyde 3-phosphate dehydrogenase (*gadph*).

**Table 2:** Number of OTUs and alpha diversity indexes of bacterial communities in the anterior (A) and posterior (P) intestinal sections of *S. aurata* fed with control (Control) and *N. gaditana* (FH) diets. Mean values and standard deviation were represented.

	OTUs	Chao 1	Shannon	Simpson	Good coverage (%)
Control-A	52.57 ± 11.82	70.50 ± 19.28	2.75 ± 0.26	0.89 ± 0.02	99.98 ± 0.005
Control-P	48.57 ± 3.95	68.15 ± 18.13	2.47 ± 0.25	0.80 ± 0.03	99.99 ± 0.001
FH-A	48.29 ± 5.79	58.75 ± 11.85	2.12 ± 0.45	0.87 ± 0.11	99.98 ± 0.007
FH-P	47.14 ± 5.25	65.75 ± 13.74	2.54 ± 0.21	0.88 ± 0.02	99.99 ± 0.004

The qPCR reactions were carried out in a C1000 Touch™ thermal cycler (BioRad, Spain) with the CFX96™ optical module (BioRad, Spain) for fluorescence measurements. Amplification reactions were performed in triplicate in 96-well plates in a final volume of 10 μL. The mixture contained 5 μL of GoTaq^®^ qPCR Master Mix (PROMEGA), 0.5 μL of forward and reverse primers (10 μM), 1 μL of cDNA and 3 μL of nuclease-free water. For the permeability and integrity genes, an initial activation of Taq polymerase at 95 °C for 3 min was used, followed by 40 cycles of 95 °C for 15 s and 60 °C for 60 s. For the immune and mucins related genes (*tnf-α*, *cox2*, *imuc*, *muc2* and *pept1*), the PCR was performed using 95 °C for 10 min, and 40 cycles of 95 °C for 10 s and 60 °C for 20 s. Finally, in the case of the reference genes (*ef1α* and *gadph*) the reactions were incubated 90 °C for 5 min followed by 40 cycles of 95 °C for 10 s, 60 °C for 10 s and 72 °C for 15 s. Threshold amplification values (Cq) greater than 40 were considered negative. Relative expression of mRNA was calculated using the method 2^(−ΔΔCq)^^82^, normalizing with a geometric average of the two reference genes and in relation to the fish of each control group. The primer sequences used to analyze gene expression in *S. aurata* were obtained from previous studies^40,64,69,83^.

Statistical analysis was performed using XLSTAT v2014.5.03 software (Addinsoft, New York, NY, USA). Results are shown as means of fold change (2^(−ΔΔCq)^) ± standard deviation (SD). The normality and homogeneity of the data were previously evaluated using the Shapiro–Wilk and Levene tests, respectively. The existence of statistically significant differences in the RT-qPCR values between the control and FH groups was determined by One-Way Analysis of Variance (ANOVA), applying the Tukey post-hoc test. When normality of the data could not be assumed, non-normal data were logarithmic transformed, and the non-parametric Kruskal–Wallis test was performed. The differences were considered statistically significant assuming *p* < 0.05.

### Ethics approval

Fish were kept and handled following the guidelines for experimental procedures in animal research from the Ethics and Animal Welfare Committee of the University of Cadiz, according to the Spanish (RD53/2013) and European Union (2012/707/EU) legislation. The Ethical Committee from the Autonomous Andalusian Government approved the experiments (Junta de Andalucía reference number 01/04/2019/047).

### Declaration ARRIVAL 2.0 protocol

This study is reported in accordance with ARRIVE guidelines (https://arriveguidelines.org).

### Consent for publication

All the authors read and agree to the content of this paper and its publication.

## Supplementary Information

Supplementary Table 1.

Supplementary Table 2.

## Data Availability

Raw read sequences of the 16S rRNA gene from *S. aurata* gut microbiota in this study are publicly available in the NCBI SRA depository within BioProject PRJNA700500, with BioSample accession numbers SAMN17721432-SAMN17721459.

## Abbreviations

OTU: Operational taxonomic unit
PCA: Principal Component Analysis
KEGG: Kyoto encyclopedia of genes and genomes
STAMP: Statistical Analysis of Metagenomics Profiles
qPCR: Quantitative PCR
SCFA: Short-chain fatty acid
