# Supplementary material for: Microbiota composition and intestinal integrity remain unaltered after the inclusion of hydrolysed Nannochloropsis gaditana in Sparus aurata diet
Source: Sci Rep. 2021 Sep 21;11:18779. doi: 10.1038/s41598-021-98087-5 (PMC8455595; doi:10.1038/s41598-021-98087-5)
Supplement: Supplementary file 1 — Supplementary Table 1. [file 41598_2021_98087_MOESM1_ESM.docx]

Supplementary table 1. List of *Sparus aurata* genes studied in this work.

| **Gene** | **Code** | | | | **Reference** |
| --- | --- | --- | --- | --- | --- |
| **Reference genes** |  | | | |  |
| Elongation factor 1α | *ef1α* | | | | (Kortner *et al.*, 2011) |
| Ribosomal glyceraldehyde 3-phosphate dehydrogenase | *gadph* | | | | (Kortner *et al.*, 2011) |
| **Permeability and integrity** |  | | | |  |
| Cadherin 1 | *cdh1* | | | | (Pérez-Sánchez *et al.*, 2015) |
| Cadherin 17 | *cdh17* | | | | (Pérez-Sánchez *et al.*, 2015) |
| Claudin 12 | *cldn12* | | | | (Pérez-Sánchez *et al.*, 2015) |
| Claudin 15 | *cldn15* | | | | (Pérez-Sánchez *et al.*, 2015) |
| Vimentin | *vim* | | | | (Pérez-Sánchez *et al.*, 2015) |
| Integrin 6-β | *itgb6* | | | | (Pérez-Sánchez *et al.*, 2015) |
| Ocludin | *ocln* | | | | (Pérez-Sánchez *et al.*, 2015) |
| Tubulin | *tub* | | | | (Cerezuela *et al*., 2013) |
| Zona-occludens 1 | *zo1* | | | | (Cerezuela *et al*., 2013) |
| **Pro-inflamatory and mucins** |  | | | |  |
| Tumoral necrosis factor α | *tnf-α* | | | | (Estruch *et al.*, 2018) |
| Cyclooxygenase 2 | *cox2* | | | | (Estruch *et al.*, 2018) |
| Intestinal mucin | *imuc* | | | | (Estruch *et al.*, 2018) |
| Mucin 2 | *muc2* | | | | (Estruch *et al.*, 2018) |
| **Nutrient absorption** | |  |  |  |  |
| Peptide transporter 1 | *pept1* | | | | (Estruch *et al.*, 2018) |
|  |  | | |  |  |
