# Supplementary material for: Microbiota composition and intestinal integrity remain unaltered after the inclusion of hydrolysed Nannochloropsis gaditana in Sparus aurata diet
Source: Sci Rep. 2021 Sep 21;11:18779. doi: 10.1038/s41598-021-98087-5 (PMC8455595; doi:10.1038/s41598-021-98087-5)
Supplement: Supplementary file 2 — Supplementary Table 2. [file 41598_2021_98087_MOESM2_ESM.docx]

Supplementary table 2. The relative abundance values of Family and Genera in each intestinal section and fish analyzed.

| **Family (Anterior)** | **C1A** | **C2A** | **C3A** | **C4A** | **C5A** | **C6A** | **C7A** | **FH1A** | **FH2A** | **FH3A** | **FH4A** | **FH5A** | **FH6A** | **FH7A** |
| --- | --- | --- | --- | --- | --- | --- | --- | --- | --- | --- | --- | --- | --- | --- |
| k__Bacteria_unclassified | 1.87 | 0.00 | 14.14 | 1.13 | 1.57 | 1.46 | 1.23 | 0.00 | 0.00 | 1.13 | 0.00 | 0.00 | 0.00 | 0.00 |
| *f__Enterobacteriaceae* | 0.00 | 0.00 | 3.16 | 0.00 | 1.68 | 0.00 | 3.77 | 0.00 | 0.00 | 51.64 | 0.00 | 0.00 | 3.00 | 0.00 |
| *o__Clostridiales_unclassified* | 1.48 | 0.00 | 0.00 | 0.00 | 0.00 | 0.00 | 0.00 | 0.00 | 0.00 | 0.00 | 0.00 | 0.00 | 0.00 | 0.00 |
| *c__Betaproteobacteria_unclassified* | 2.55 | 1.63 | 1.14 | 2.64 | 0.00 | 0.00 | 0.00 | 1.58 | 0.00 | 0.00 | 0.00 | 0.00 | 1.16 | 1.12 |
| *f__Pseudomonadaceae* | 7.29 | 1.95 | 3.91 | 13.79 | 3.85 | 0.00 | 2.49 | 8.78 | 8.16 | 2.45 | 0.00 | 5.19 | 17.28 | 0.00 |
| *f__Tissierellaceae* | 0.00 | 0.00 | 0.00 | 6.75 | 0.00 | 6.29 | 0.00 | 0.00 | 0.00 | 0.00 | 0.00 | 0.00 | 0.00 | 0.00 |
| *f__Corynebacteriaceae* | 8.76 | 4.24 | 7.33 | 3.93 | 1.94 | 0.00 | 2.46 | 0.00 | 6.75 | 0.00 | 0.00 | 1.26 | 2.78 | 11.37 |
| *f__Bradyrhizobiaceae* | 0.00 | 3.69 | 0.00 | 0.00 | 0.00 | 0.00 | 1.34 | 0.00 | 0.00 | 0.00 | 0.00 | 0.00 | 0.00 | 0.00 |
| *f__Oxalobacteraceae* | 4.73 | 0.00 | 0.00 | 0.00 | 3.10 | 0.00 | 0.00 | 0.00 | 0.00 | 0.00 | 0.00 | 0.00 | 0.00 | 0.00 |
| *f__Micrococcaceae* | 0.00 | 0.00 | 0.00 | 0.00 | 1.84 | 0.00 | 0.00 | 11.65 | 0.00 | 2.98 | 1.13 | 1.06 | 1.00 | 1.20 |
| *f__Rhodobacteraceae* | 0.00 | 0.00 | 0.00 | 0.00 | 1.90 | 0.00 | 0.00 | 0.00 | 0.00 | 0.00 | 0.00 | 0.00 | 0.00 | 2.04 |
| *f__Veillonellaceae* | 6.90 | 0.00 | 1.30 | 0.00 | 2.03 | 0.00 | 2.01 | 0.00 | 0.00 | 0.00 | 0.00 | 0.00 | 4.57 | 0.00 |
| *f__Sphingomonadaceae* | 0.00 | 8.89 | 0.00 | 0.00 | 0.00 | 6.96 | 12.34 | 0.00 | 0.00 | 2.04 | 0.00 | 9.69 | 0.00 | 24.53 |
| *f__Moraxellaceae* | 0.00 | 0.00 | 5.06 | 1.30 | 4.45 | 1.90 | 13.23 | 0.00 | 10.87 | 6.00 | 1.42 | 0.00 | 0.00 | 0.00 |
| *f__Methylobacteriaceae* | 0.00 | 0.00 | 0.00 | 0.00 | 1.11 | 2.06 | 0.00 | 2.26 | 0.00 | 0.00 | 0.00 | 0.00 | 2.82 | 0.00 |
| *f__Vibrionaceae* | 0.00 | 0.00 | 0.00 | 1.83 | 0.00 | 0.00 | 0.00 | 0.00 | 0.00 | 0.00 | 0.00 | 0.00 | 0.00 | 0.00 |
| *f__Shewanellaceae* | 0.00 | 4.82 | 4.29 | 1.39 | 6.22 | 0.00 | 0.00 | 4.64 | 0.00 | 0.00 | 0.00 | 1.68 | 0.00 | 0.00 |
| *f__Pasteurellaceae* | 0.00 | 8.86 | 8.64 | 0.00 | 0.00 | 0.00 | 0.00 | 0.00 | 0.00 | 0.00 | 0.00 | 4.36 | 0.00 | 5.03 |
| *o__Flavobacteriales_unclassified* | 0.00 | 0.00 | 0.00 | 0.00 | 0.00 | 0.00 | 0.00 | 0.00 | 0.00 | 0.00 | 0.00 | 0.00 | 0.00 | 1.05 |
| *f__Clostridiaceae* | 7.44 | 0.00 | 0.00 | 0.00 | 0.00 | 0.00 | 0.00 | 0.00 | 0.00 | 0.00 | 0.00 | 0.00 | 0.00 | 0.00 |
| *f__Propionibacteriaceae* | 13.15 | 12.98 | 9.39 | 12.94 | 4.12 | 8.27 | 16.08 | 9.39 | 16.94 | 7.82 | 2.13 | 12.41 | 4.76 | 15.74 |
| *f__Actinomycetaceae* | 0.00 | 0.00 | 0.00 | 2.99 | 0.00 | 0.00 | 1.70 | 0.00 | 0.00 | 0.00 | 0.00 | 0.00 | 0.00 | 0.00 |
| *f__Comamonadaceae* | 5.76 | 5.35 | 3.43 | 4.44 | 0.00 | 2.18 | 2.31 | 4.42 | 0.00 | 0.00 | 0.00 | 0.00 | 8.54 | 3.75 |
| *f__Xanthomonadaceae* | 0.00 | 0.00 | 0.00 | 0.00 | 4.44 | 0.00 | 0.00 | 0.00 | 0.00 | 0.00 | 0.00 | 0.00 | 0.00 | 0.00 |
| *o__Actinomycetales_unclassified* | 13.25 | 12.11 | 2.18 | 14.39 | 17.91 | 26.03 | 10.32 | 7.16 | 14.48 | 6.08 | 34.56 | 24.63 | 22.31 | 6.66 |
| *o__Vibrionales_unclassified* | 2.03 | 5.07 | 3.21 | 0.00 | 9.62 | 1.13 | 2.72 | 0.00 | 5.98 | 0.00 | 0.00 | 15.06 | 6.67 | 0.00 |
| *f__* | 6.38 | 16.66 | 0.00 | 0.00 | 5.66 | 9.01 | 6.29 | 0.00 | 6.16 | 0.00 | 0.00 | 0.00 | 0.00 | 20.77 |
| *o__Bacillales_unclassified* | 0.00 | 0.00 | 0.00 | 1.69 | 3.20 | 1.34 | 0.00 | 2.27 | 1.03 | 1.07 | 1.83 | 1.89 | 1.45 | 2.21 |
| *f__Gemellaceae* | 0.00 | 0.00 | 0.00 | 11.41 | 0.00 | 0.00 | 0.00 | 0.00 | 0.00 | 0.00 | 0.00 | 0.00 | 0.00 | 0.00 |
| *f__Fusobacteriaceae* | 0.00 | 0.00 | 0.00 | 0.00 | 0.00 | 0.00 | 0.00 | 7.50 | 0.00 | 0.00 | 0.00 | 0.00 | 0.00 | 0.00 |
| *f__Carnobacteriaceae* | 0.00 | 0.00 | 0.00 | 6.20 | 0.00 | 0.00 | 0.00 | 0.00 | 0.00 | 0.00 | 0.00 | 0.00 | 0.00 | 0.00 |
| *c__Alphaproteobacteria_unclassified* | 0.00 | 0.00 | 0.00 | 0.00 | 0.00 | 0.00 | 0.00 | 0.00 | 0.00 | 0.00 | 0.00 | 0.00 | 0.00 | 0.00 |
| *f__Alcaligenaceae* | 0.00 | 4.66 | 1.59 | 5.82 | 2.41 | 0.00 | 0.00 | 1.09 | 0.00 | 0.00 | 0.00 | 2.32 | 0.00 | 0.00 |
| *c__Bacilli_unclassified* | 13.59 | 8.22 | 29.31 | 6.45 | 19.44 | 25.52 | 17.95 | 38.06 | 27.39 | 15.55 | 56.12 | 18.16 | 23.57 | 3.52 |
| *c__Gammaproteobacteria_unclassified* | 3.97 | 0.00 | 0.00 | 0.00 | 1.10 | 5.94 | 0.00 | 0.00 | 0.00 | 0.00 | 0.00 | 0.00 | 0.00 | 0.00 |
| ETC (<1%) | 0.84 | 0.57 | 1.91 | 0.91 | 2.41 | 1.91 | 3.55 | 1.20 | 2.23 | 3.23 | 2.81 | 2.29 | 0.06 | 1.00 |

| **Family (posterior)** | **C1P** | **C2P** | **C3P** | **C4P** | **C5P** | **C6P** | **C7P** | **FH1P** | **FH2P** | **FH3P** | **FH4P** | **FH5P** | **FH6P** |
| --- | --- | --- | --- | --- | --- | --- | --- | --- | --- | --- | --- | --- | --- |
| *k__Bacteria_unclassified* | 0.00 | 0.00 | 36.43 | 6.13 | 0.00 | 0.00 | 1.09 | 1.03 | 0.00 | 0.00 | 0.00 | 0.00 | 0.00 |
| *o__Bacteroidales_unclassified* | 0.00 | 0.00 | 0.00 | 0.00 | 0.00 | 0.00 | 0.00 | 0.00 | 0.00 | 0.00 | 0.00 | 0.00 | 0.00 |
| *f__Enterobacteriaceae* | 0.00 | 0.00 | 4.82 | 6.13 | 1.54 | 0.00 | 0.00 | 3.03 | 2.65 | 1.35 | 0.00 | 0.00 | 8.14 |
| *o__Clostridiales_unclassified* | 0.00 | 0.00 | 0.00 | 0.00 | 8.11 | 0.00 | 0.00 | 0.00 | 0.00 | 0.00 | 0.00 | 0.00 | 0.00 |
| *c__Betaproteobacteria_unclassified* | 2.43 | 0.00 | 0.00 | 1.01 | 0.00 | 0.00 | 1.00 | 0.00 | 4.81 | 3.30 | 0.00 | 0.00 | 0.00 |
| *f__Pseudomonadaceae* | 9.32 | 0.00 | 4.41 | 1.94 | 9.44 | 13.14 | 6.09 | 6.97 | 4.42 | 0.00 | 15.99 | 1.20 | 0.00 |
| *f__Tissierellaceae* | 7.05 | 0.00 | 0.00 | 0.00 | 0.00 | 0.00 | 0.00 | 0.00 | 6.21 | 0.00 | 0.00 | 4.69 | 14.45 |
| *f__Oceanospirillaceae* | 0.00 | 0.00 | 0.00 | 0.00 | 0.00 | 0.00 | 0.00 | 0.00 | 0.00 | 3.46 | 0.00 | 0.00 | 0.00 |
| *f__Corynebacteriaceae* | 0.00 | 5.61 | 1.34 | 9.25 | 1.56 | 7.99 | 4.56 | 2.01 | 7.33 | 3.96 | 5.34 | 3.18 | 7.35 |
| *f__Bradyrhizobiaceae* | 3.36 | 0.00 | 0.00 | 0.00 | 0.00 | 3.92 | 0.00 | 0.00 | 0.00 | 0.00 | 0.00 | 0.00 | 0.00 |
| *f__Oxalobacteraceae* | 0.00 | 0.00 | 1.30 | 0.00 | 1.95 | 0.00 | 0.00 | 8.05 | 0.00 | 1.07 | 0.00 | 2.58 | 0.00 |
| *f__Micrococcaceae* | 0.00 | 0.00 | 0.00 | 0.00 | 0.00 | 0.00 | 0.00 | 3.72 | 2.96 | 0.00 | 1.90 | 2.00 | 2.00 |
| *f__Rhodobacteraceae* | 0.00 | 0.00 | 0.00 | 0.00 | 4.17 | 0.00 | 0.00 | 0.00 | 1.24 | 0.00 | 0.00 | 0.00 | 0.00 |
| *f__Veillonellaceae* | 0.00 | 0.00 | 0.00 | 0.00 | 0.00 | 0.00 | 0.00 | 0.00 | 0.00 | 4.74 | 0.00 | 0.00 | 0.00 |
| *f__Sphingomonadaceae* | 10.81 | 15.52 | 0.00 | 0.00 | 7.35 | 0.00 | 0.00 | 0.00 | 0.00 | 0.00 | 2.26 | 12.69 | 0.00 |
| *f__Moraxellaceae* | 0.00 | 9.39 | 1.42 | 0.00 | 0.00 | 0.00 | 0.00 | 7.06 | 0.00 | 0.00 | 0.00 | 0.00 | 0.00 |
| *f__Methylobacteriaceae* | 0.00 | 1.85 | 0.00 | 0.00 | 0.00 | 0.00 | 1.48 | 0.00 | 0.00 | 0.00 | 0.00 | 0.00 | 0.00 |
| *f__Vibrionaceae* | 1.34 | 0.00 | 0.00 | 0.00 | 0.00 | 0.00 | 1.25 | 0.00 | 0.00 | 0.00 | 0.00 | 3.79 | 0.00 |
| *f__Shewanellaceae* | 0.00 | 0.00 | 0.00 | 0.00 | 4.30 | 0.00 | 2.65 | 2.79 | 1.55 | 0.00 | 0.00 | 0.00 | 2.44 |
| *f__Pasteurellaceae* | 0.00 | 0.00 | 1.63 | 9.65 | 4.13 | 0.00 | 0.00 | 0.00 | 2.69 | 0.00 | 0.00 | 0.00 | 0.00 |
| *f__Clostridiaceae* | 0.00 | 0.00 | 6.26 | 0.00 | 0.00 | 0.00 | 0.00 | 0.00 | 0.00 | 0.00 | 0.00 | 10.25 | 0.00 |
| *f__Propionibacteriaceae* | 14.60 | 23.71 | 5.39 | 12.79 | 9.98 | 14.15 | 3.73 | 8.00 | 21.95 | 19.51 | 7.98 | 12.59 | 8.44 |
| *f__Comamonadaceae* | 6.24 | 3.21 | 1.22 | 3.16 | 4.47 | 0.00 | 2.79 | 0.00 | 4.76 | 9.76 | 1.69 | 2.16 | 1.46 |
| *f__Xanthomonadaceae* | 0.00 | 0.00 | 0.00 | 0.00 | 0.00 | 0.00 | 0.00 | 0.00 | 0.00 | 0.00 | 3.54 | 0.00 | 0.00 |
| *o__Actinomycetales_unclassified* | 5.70 | 0.00 | 16.26 | 19.83 | 10.30 | 12.55 | 20.96 | 16.05 | 12.94 | 16.45 | 20.79 | 21.81 | 17.88 |
| *o__Vibrionales_unclassified* | 3.26 | 0.00 | 0.00 | 0.00 | 9.99 | 8.14 | 14.53 | 3.95 | 3.59 | 0.00 | 0.00 | 8.56 | 4.69 |
| *f__* | 5.91 | 13.83 | 0.00 | 3.91 | 1.80 | 5.16 | 13.28 | 5.07 | 0.00 | 0.00 | 7.44 | 0.00 | 0.00 |
| *o__Bacillales_unclassified* | 1.18 | 0.00 | 0.00 | 0.00 | 4.63 | 0.00 | 0.00 | 1.87 | 1.14 | 2.21 | 1.02 | 0.00 | 0.00 |
| *f__Gemellaceae* | 0.00 | 0.00 | 3.28 | 0.00 | 0.00 | 0.00 | 0.00 | 0.00 | 0.00 | 4.23 | 0.00 | 0.00 | 0.00 |
| *f__Fusobacteriaceae* | 0.00 | 0.00 | 0.00 | 1.79 | 0.00 | 0.00 | 0.00 | 0.00 | 0.00 | 0.00 | 0.00 | 0.00 | 0.00 |
| *f__Carnobacteriaceae* | 0.00 | 4.11 | 0.00 | 5.03 | 0.00 | 0.00 | 0.00 | 0.00 | 0.00 | 0.00 | 0.00 | 0.00 | 0.00 |
| *f__Alcaligenaceae* | 1.70 | 0.00 | 0.00 | 0.00 | 0.00 | 0.00 | 4.49 | 8.02 | 0.00 | 0.00 | 0.00 | 1.88 | 3.28 |
| *c__Bacilli_unclassified* | 25.09 | 19.23 | 14.53 | 18.70 | 13.69 | 30.78 | 20.65 | 20.69 | 20.86 | 28.02 | 29.35 | 12.51 | 27.63 |
| *ETC (<1%)* | 2.01 | 3.54 | 1.70 | 0.68 | 2.59 | 4.17 | 1.44 | 1.69 | 0.89 | 1.95 | 2.71 | 0.11 | 2.22 |

| **Genera (Anterior)** | **C1A** | **C2A** | **C3A** | **C4A** | **C5A** | **C6A** | **C7A** | **FH1A** | **FH2A** | **FH3A** | **FH4A** | **FH5A** | **FH6A** | **FH7A** |
| --- | --- | --- | --- | --- | --- | --- | --- | --- | --- | --- | --- | --- | --- | --- |
| *k__Bacteria_unclassified* | 1.87 | 0.00 | 4.14 | 1.13 | 1.07 | 1.46 | 1.23 | 0.00 | 0.00 | 1.13 | 1.13 | 1.06 | 0.00 | 0.00 |
| *f__Enterobacteriaceae_unclassified* | 0.00 | 0.00 | 0.00 | 0.00 | 1.18 | 0.00 | 0.00 | 0.00 | 0.00 | 6.05 | 0.00 | 0.00 | 3.00 | 0.00 |
| *o__Clostridiales_unclassified* | 1.48 | 10.00 | 10.09 | 1.10 | 2.00 | 7.99 | 8.00 | 0.00 | 0.00 | 0.00 | 0.00 | 0.00 | 0.00 | 0.00 |
| *c__Betaproteobacteria_unclassified* | 2.55 | 1.63 | 1.14 | 2.64 | 0.00 | 0.00 | 0.00 | 1.58 | 0.00 | 0.00 | 0.00 | 0.00 | 1.16 | 1.12 |
| *f__Pseudomonadaceae_unclassified* | 7.29 | 1.95 | 3.91 | 12.79 | 3.85 | 0.00 | 2.49 | 8.78 | 8.16 | 2.45 | 0.00 | 5.19 | 17.28 | 0.00 |
| *g__* | 6.38 | 6.66 | 2.86 | 0.00 | 3.66 | 1.05 | 1.62 | 0.00 | 6.16 | 45.66 | 0.00 | 0.00 | 0.00 | 21.79 |
| *g__Anaerococcus* | 5.00 | 2.00 | 3.03 | 6.75 | 1.00 | 1.00 | 10.00 | 0.00 | 0.00 | 0.00 | 0.00 | 0.00 | 0.00 | 0.00 |
| *g__Corynebacterium* | 3.76 | 2.24 | 4.30 | 3.93 | 1.00 | 0.00 | 2.46 | 0.00 | 6.75 | 0.00 | 0.00 | 1.26 | 2.78 | 11.37 |
| *f__Bradyrhizobiaceae_unclassified* | 0.00 | 2.18 | 0.00 | 0.00 | 0.00 | 0.00 | 0.00 | 0.00 | 0.00 | 0.00 | 0.00 | 0.00 | 0.00 | 0.00 |
| *f__Micrococcaceae_unclassified* | 0.00 | 4.15 | 0.00 | 0.00 | 0.00 | 0.00 | 2.57 | 0.00 | 0.00 | 0.00 | 0.00 | 0.00 | 0.00 | 0.00 |
| *f__Rhodobacteraceae_unclassified* | 0.00 | 0.00 | 0.00 | 0.00 | 0.00 | 0.00 | 0.00 | 0.00 | 0.00 | 0.00 | 0.00 | 0.00 | 0.00 | 2.04 |
| *g__Sphingomonas* | 0.00 | 8.89 | 0.00 | 0.00 | 0.00 | 6.06 | 2.34 | 0.00 | 0.00 | 2.04 | 0.00 | 9.69 | 0.00 | 24.53 |
| *g__Enhydrobacter* | 0.00 | 0.00 | 3.97 | 0.00 | 0.00 | 0.00 | 0.00 | 0.00 | 0.00 | 0.00 | 0.00 | 0.00 | 0.00 | 0.00 |
| *g__Methylobacterium* | 0.00 | 0.00 | 0.00 | 0.00 | 1.11 | 2.03 | 0.00 | 2.26 | 0.00 | 0.00 | 0.00 | 0.00 | 2.78 | 0.00 |
| *g__Vibrio* | 0.00 | 0.00 | 0.00 | 1.83 | 0.00 | 0.00 | 0.00 | 0.00 | 0.00 | 0.00 | 0.00 | 0.00 | 0.00 | 0.00 |
| *g__Shewanella* | 0.00 | 4.82 | 4.29 | 1.39 | 6.22 | 0.00 | 0.00 | 4.64 | 0.00 | 0.00 | 0.00 | 1.68 | 0.00 | 0.00 |
| *g__Haemophilus* | 0.00 | 8.29 | 6.93 | 0.00 | 0.00 | 0.00 | 0.00 | 0.00 | 0.00 | 0.00 | 0.00 | 4.08 | 0.00 | 4.78 |
| *g__Finegoldia* | 0.00 | 0.00 | 0.00 | 0.00 | 0.00 | 6.28 | 0.00 | 0.00 | 0.00 | 0.00 | 0.00 | 0.00 | 0.00 | 0.00 |
| *o__Flavobacteriales_unclassified* | 0.00 | 0.00 | 0.00 | 0.00 | 0.00 | 0.00 | 0.00 | 0.00 | 0.00 | 0.00 | 0.00 | 0.00 | 0.00 | 1.05 |
| *g__Candidatus_Arthromitus* | 7.44 | 0.00 | 0.00 | 0.00 | 0.00 | 0.00 | 0.00 | 0.00 | 0.00 | 0.00 | 0.00 | 0.00 | 0.00 | 0.00 |
| *g__Propionibacterium* | 13.15 | 12.98 | 9.39 | 12.94 | 4.12 | 8.27 | 6.08 | 19.39 | 16.94 | 7.82 | 2.13 | 12.41 | 4.76 | 15.74 |
| *g__Kocuria* | 0.00 | 0.00 | 0.00 | 0.00 | 0.00 | 0.00 | 1.55 | 0.00 | 0.00 | 0.00 | 0.00 | 0.00 | 0.00 | 0.00 |
| *g__Actinomyces* | 0.00 | 0.00 | 0.00 | 2.99 | 0.00 | 0.00 | 1.70 | 0.00 | 0.00 | 0.00 | 0.00 | 0.00 | 0.00 | 0.00 |
| *g__Rothia* | 0.00 | 2.17 | 0.00 | 0.00 | 0.00 | 0.00 | 6.10 | 0.00 | 0.00 | 2.30 | 0.00 | 0.00 | 0.00 | 0.00 |
| *g__Janthinobacterium* | 4.47 | 0.00 | 0.00 | 0.00 | 2.68 | 0.00 | 0.00 | 0.00 | 0.00 | 0.00 | 0.00 | 0.00 | 0.00 | 0.00 |
| *g__Paracoccus* | 0.00 | 0.00 | 0.00 | 0.00 | 1.45 | 0.00 | 0.00 | 0.00 | 0.00 | 0.00 | 0.00 | 0.00 | 0.00 | 0.00 |
| *g__Acinetobacter* | 0.00 | 0.00 | 1.09 | 1.30 | 4.45 | 1.90 | 13.23 | 0.00 | 10.87 | 6.00 | 1.42 | 0.00 | 0.00 | 0.00 |
| *g__Stenotrophomonas* | 0.00 | 0.00 | 0.00 | 0.00 | 4.44 | 0.00 | 0.00 | 0.00 | 0.00 | 0.00 | 0.00 | 0.00 | 0.00 | 0.00 |
| *o__Actinomycetales_unclassified* | 13.25 | 6.11 | 2.18 | 14.39 | 17.91 | 26.03 | 10.32 | 7.16 | 14.48 | 6.08 | 34.56 | 24.63 | 22.31 | 6.66 |
| *g__Pelomonas* | 5.75 | 5.35 | 3.43 | 4.44 | 1.00 | 1.75 | 2.31 | 4.42 | 0.00 | 0.00 | 0.00 | 0.00 | 2.32 | 3.75 |
| *o__Vibrionales_unclassified* | 2.03 | 5.07 | 3.21 | 0.00 | 9.62 | 1.13 | 2.72 | 0.00 | 5.98 | 0.00 | 0.00 | 15.06 | 6.67 | 0.00 |
| *o__Bacillales_unclassified* | 0.00 | 0.00 | 0.00 | 1.69 | 3.20 | 1.34 | 0.00 | 2.27 | 1.03 | 1.07 | 1.83 | 1.89 | 1.45 | 2.21 |
| *f__Gemellaceae_unclassified* | 0.00 | 0.00 | 0.00 | 11.40 | 0.00 | 0.00 | 0.00 | 0.00 | 0.00 | 0.00 | 0.00 | 0.00 | 0.00 | 0.00 |
| *f__Fusobacteriaceae_unclassified* | 0.00 | 0.00 | 0.00 | 0.00 | 0.00 | 0.00 | 0.00 | 7.50 | 0.00 | 0.00 | 0.00 | 0.00 | 0.00 | 0.00 |
| *g__Afipia* | 0.00 | 1.50 | 0.00 | 0.00 | 0.00 | 0.00 | 0.00 | 0.00 | 0.00 | 0.00 | 0.00 | 0.00 | 0.00 | 0.00 |
| *g__Granulicatella* | 0.00 | 0.00 | 0.00 | 6.20 | 0.00 | 0.00 | 0.00 | 0.00 | 0.00 | 0.00 | 0.00 | 0.00 | 0.00 | 0.00 |
| *g__Curvibacter* | 0.00 | 0.00 | 0.00 | 0.00 | 0.00 | 0.00 | 0.00 | 0.00 | 0.00 | 0.00 | 0.00 | 0.00 | 6.22 | 0.00 |
| *g__Achromobacter* | 0.00 | 4.66 | 1.59 | 5.82 | 2.41 | 0.00 | 0.00 | 1.09 | 0.00 | 0.00 | 0.00 | 0.00 | 0.00 | 0.00 |
| *f__Lachnospiraceae_unclassified* | 0.00 | 0.00 | 0.00 | 0.00 | 0.00 | 0.00 | 0.00 | 0.00 | 0.00 | 0.00 | 0.00 | 2.32 | 0.00 | 0.00 |
| *c__Bacilli_unclassified* | 13.59 | 8.22 | 29.31 | 6.45 | 19.44 | 25.52 | 17.95 | 38.06 | 27.39 | 15.55 | 56.12 | 18.16 | 23.57 | 3.52 |
| *g__Actinobacillus* | 0.00 | 0.00 | 1.16 | 0.00 | 0.00 | 0.00 | 0.00 | 0.00 | 0.00 | 0.00 | 0.00 | 0.00 | 0.00 | 0.00 |
| *g__Veillonella* | 6.90 | 0.00 | 1.30 | 0.00 | 2.02 | 0.00 | 2.00 | 0.00 | 0.00 | 0.00 | 0.00 | 0.00 | 4.57 | 0.00 |
| *c__Gammaproteobacteria_unclassified* | 3.97 | 0.00 | 0.00 | 0.00 | 1.10 | 5.94 | 0.00 | 0.00 | 0.00 | 0.00 | 0.00 | 0.00 | 0.00 | 0.00 |
| ETC (<1%) | 1.10 | 1.13 | 2.77 | 0.91 | 5.12 | 2.35 | 5.34 | 2.85 | 2.23 | 3.84 | 2.81 | 2.57 | 1.12 | 1.43 |

| **Genera (Posterior)** | **C1P** | **C2P** | **C3P** | **C4P** | **C5P** | **C6P** | **C7P** | **FH1P** | **FH2P** | **FH3P** | **FH4P** | **FH5P** | **FH6P** | **FH7P** |
| --- | --- | --- | --- | --- | --- | --- | --- | --- | --- | --- | --- | --- | --- | --- |
| *k__Bacteria_unclassified* | 0.00 | 0.00 | 36.43 | 6.13 | 0.00 | 0.00 | 1.09 | 1.03 | 0.00 | 0.00 | 0.00 | 0.00 | 0.00 | 1.44 |
| *f__Enterobacteriaceae_unclassified* | 0.00 | 0.00 | 4.82 | 6.13 | 0.00 | 0.00 | 0.00 | 3.03 | 2.65 | 1.34 | 0.00 | 0.00 | 4.46 | 0.00 |
| *o__Clostridiales_unclassified* | 0.00 | 0.00 | 0.00 | 0.00 | 8.11 | 0.00 | 0.00 | 0.00 | 0.00 | 0.00 | 0.00 | 0.00 | 0.00 | 0.00 |
| *c__Betaproteobacteria_unclassified* | 2.43 | 0.00 | 0.00 | 1.01 | 0.00 | 0.00 | 1.00 | 0.00 | 4.81 | 3.30 | 0.00 | 0.00 | 0.00 | 0.00 |
| *f__Pseudomonadaceae_unclassified* | 9.32 | 0.00 | 4.19 | 1.94 | 9.42 | 13.14 | 6.09 | 6.97 | 4.42 | 0.00 | 15.99 | 1.20 | 0.00 | 5.40 |
| *g__* | 5.91 | 13.83 | 0.00 | 3.91 | 3.14 | 5.16 | 14.75 | 5.08 | 0.00 | 0.00 | 7.44 | 0.00 | 3.96 | 3.50 |
| *g__Anaerococcus* | 0.00 | 0.00 | 0.00 | 0.00 | 0.00 | 0.00 | 0.00 | 0.00 | 6.21 | 0.00 | 0.00 | 0.00 | 0.00 | 0.00 |
| *g__Corynebacterium* | 0.00 | 5.61 | 1.34 | 9.25 | 1.56 | 7.99 | 4.56 | 2.01 | 4.33 | 3.96 | 5.34 | 2.18 | 7.35 | 5.79 |
| *f__Bradyrhizobiaceae_unclassified* | 1.89 | 0.00 | 0.00 | 0.00 | 0.00 | 2.41 | 0.00 | 0.00 | 0.00 | 0.00 | 0.00 | 0.00 | 0.00 | 0.00 |
| *f__Micrococcaceae_unclassified* | 0.00 | 0.00 | 0.00 | 0.00 | 0.00 | 0.00 | 0.00 | 2.18 | 0.00 | 0.00 | 0.00 | 0.00 | 0.00 | 0.00 |
| *f__Rhodobacteraceae_unclassified* | 0.00 | 0.00 | 0.00 | 0.00 | 4.08 | 0.00 | 0.00 | 0.00 | 0.00 | 0.00 | 0.00 | 0.00 | 0.00 | 0.00 |
| *g__Sphingomonas* | 10.81 | 15.52 | 0.00 | 0.00 | 7.35 | 0.00 | 0.00 | 0.00 | 0.00 | 0.00 | 2.26 | 12.69 | 0.00 | 0.00 |
| *g__Enhydrobacter* | 0.00 | 0.00 | 0.00 | 0.00 | 0.00 | 0.00 | 0.00 | 4.72 | 3.00 | 4.42 | 1.90 | 1.00 | 3.96 | 11.15 |
| *g__Methylobacterium* | 0.00 | 1.85 | 0.00 | 0.00 | 0.00 | 0.00 | 0.00 | 0.00 | 0.00 | 0.00 | 0.00 | 0.00 | 0.00 | 0.00 |
| *g__Vibrio* | 1.34 | 0.00 | 0.00 | 0.00 | 0.00 | 0.00 | 1.25 | 0.00 | 0.00 | 0.00 | 0.00 | 3.79 | 0.00 | 0.00 |
| *g__Shewanella* | 0.00 | 0.00 | 0.00 | 0.00 | 4.30 | 0.00 | 2.65 | 2.79 | 1.55 | 0.00 | 0.00 | 0.00 | 2.44 | 4.73 |
| *g__Haemophilus* | 0.00 | 0.00 | 1.51 | 4.53 | 3.99 | 0.00 | 0.00 | 0.00 | 2.46 | 0.00 | 0.00 | 0.00 | 0.00 | 0.00 |
| *g__Finegoldia* | 0.00 | 0.00 | 0.00 | 0.00 | 0.00 | 0.00 | 0.00 | 0.00 | 0.00 | 0.00 | 0.00 | 0.00 | 9.00 | 0.00 |
| *g__Candidatus_Arthromitus* | 0.00 | 0.00 | 6.26 | 0.00 | 0.00 | 0.00 | 0.00 | 0.00 | 0.00 | 0.00 | 0.00 | 10.25 | 0.00 | 5.25 |
| *g__Propionibacterium* | 14.60 | 23.71 | 5.39 | 12.79 | 9.98 | 14.15 | 3.73 | 8.00 | 21.95 | 19.51 | 7.98 | 12.59 | 8.44 | 7.81 |
| *g__Kocuria* | 0.00 | 0.00 | 0.00 | 0.00 | 0.00 | 0.00 | 0.00 | 1.55 | 2.37 | 0.00 | 0.00 | 0.00 | 0.00 | 0.00 |
| *g__Peptoniphilus* | 7.05 | 0.00 | 0.00 | 0.00 | 0.00 | 0.00 | 0.00 | 0.00 | 0.00 | 0.00 | 0.00 | 4.69 | 5.45 | 0.00 |
| *g__Schlegelella* | 0.00 | 0.00 | 0.00 | 0.00 | 3.05 | 0.00 | 0.00 | 0.00 | 0.00 | 0.00 | 0.00 | 0.00 | 0.00 | 1.91 |
| *g__Janthinobacterium* | 0.00 | 0.00 | 1.18 | 0.00 | 0.00 | 0.00 | 0.00 | 0.00 | 0.00 | 0.00 | 0.00 | 2.39 | 0.00 | 0.00 |
| *g__Paracoccus* | 0.00 | 0.00 | 0.00 | 0.00 | 0.00 | 0.00 | 0.00 | 0.00 | 1.21 | 0.00 | 0.00 | 0.00 | 0.00 | 0.00 |
| *g__Acinetobacter* | 0.00 | 9.39 | 1.42 | 0.00 | 0.00 | 0.00 | 0.00 | 2.34 | 0.00 | 0.00 | 0.00 | 0.00 | 0.00 | 4.85 |
| *g__Stenotrophomonas* | 0.00 | 0.00 | 0.00 | 0.00 | 0.00 | 0.00 | 0.00 | 0.00 | 0.00 | 0.00 | 3.54 | 0.00 | 0.00 | 0.00 |
| *o__Actinomycetales_unclassified* | 5.70 | 0.00 | 16.26 | 19.83 | 10.30 | 12.55 | 20.96 | 16.05 | 12.94 | 16.45 | 20.79 | 21.81 | 17.88 | 9.64 |
| *g__Pelomonas* | 6.24 | 2.22 | 1.22 | 3.16 | 1.41 | 0.00 | 2.79 | 0.00 | 4.76 | 9.76 | 1.66 | 2.16 | 1.46 | 1.59 |
| *o__Vibrionales_unclassified* | 3.26 | 0.00 | 0.00 | 0.00 | 9.99 | 8.14 | 14.53 | 3.95 | 3.59 | 0.00 | 0.00 | 8.56 | 4.69 | 3.87 |
| *o__Bacillales_unclassified* | 1.18 | 0.00 | 0.00 | 0.00 | 4.63 | 0.00 | 0.00 | 1.87 | 1.14 | 2.21 | 1.02 | 0.00 | 0.00 | 0.00 |
| *f__Gemellaceae_unclassified* | 0.00 | 0.00 | 2.60 | 0.00 | 0.00 | 0.00 | 0.00 | 0.00 | 0.00 | 3.40 | 0.00 | 0.00 | 0.00 | 0.00 |
| *f__Fusobacteriaceae_unclassified* | 0.00 | 0.00 | 0.00 | 1.79 | 0.00 | 0.00 | 0.00 | 0.00 | 0.00 | 0.00 | 0.00 | 0.00 | 0.00 | 0.00 |
| *g__Afipia* | 1.47 | 0.00 | 0.00 | 0.00 | 0.00 | 1.51 | 0.00 | 0.00 | 0.00 | 0.00 | 0.00 | 0.00 | 0.00 | 0.00 |
| *g__Granulicatella* | 0.00 | 4.11 | 0.00 | 5.03 | 0.00 | 0.00 | 0.00 | 0.00 | 0.00 | 0.00 | 0.00 | 0.00 | 0.00 | 0.00 |
| *g__Ralstonia* | 0.00 | 0.00 | 0.00 | 0.00 | 1.95 | 0.00 | 0.00 | 8.04 | 0.00 | 1.06 | 0.00 | 0.00 | 0.00 | 2.63 |
| *g__Achromobacter* | 1.70 | 0.00 | 0.00 | 0.00 | 0.00 | 0.00 | 4.49 | 8.02 | 0.00 | 0.00 | 0.00 | 1.88 | 3.28 | 0.00 |
| *c__Bacilli_unclassified* | 25.09 | 19.23 | 14.53 | 18.70 | 13.69 | 30.78 | 20.65 | 20.69 | 20.86 | 28.02 | 29.35 | 12.51 | 27.63 | 26.94 |
| *g__Actinobacillus* | 0.00 | 0.00 | 0.00 | 4.67 | 0.00 | 0.00 | 0.00 | 0.00 | 0.00 | 0.00 | 0.00 | 0.00 | 0.00 | 0.00 |
| *g__Veillonella* | 0.00 | 0.00 | 0.00 | 0.00 | 0.00 | 0.00 | 0.00 | 0.00 | 0.00 | 4.73 | 0.00 | 0.00 | 0.00 | 0.00 |
| ETC (<1%) | 2.01 | 4.53 | 2.84 | 1.14 | 3.06 | 4.16 | 1.44 | 1.70 | 1.74 | 1.84 | 2.73 | 2.30 | 3.96 | 3.49 |
